# Fungal Attachment-Resistant Polymers for the Additive Manufacture of Medical Devices

**DOI:** 10.1021/acsami.4c04833

**Published:** 2024-09-30

**Authors:** Ling Xin Yong, Joseph Sefton, Cindy Vallières, Graham A. Rance, Jordan Hill, Valentina Cuzzucoli Crucitti, Adam A. Dundas, Felicity R. A. J. Rose, Morgan R. Alexander, Ricky Wildman, Yinfeng He, Simon V. Avery, Derek J. Irvine

**Affiliations:** † Centre for Additive Manufacturing, Department of Chemical and Environmental Engineering, University of Nottingham, University Park, Nottingham NG7 2RD, United Kingdom; ‡ School of Life Sciences, University of Nottingham, University Park, Nottingham NG7 2RD, United Kingdom; § Nanoscale and Microscale Research Centre, University of Nottingham, University Park, Nottingham NG7 2RD, United Kingdom; ∥ Advanced Materials Research Group, Department of Chemical and Environmental Engineering, University of Nottingham, University Park, Nottingham NG7 2RD, United Kingdom; ⊥ School of Pharmacy, University of Nottingham, Nottingham NG7 2RD, United Kingdom

**Keywords:** polymer chemistry, fungal adherence, additive manufacturing, high-throughput screening, medical device, bioassay development

## Abstract

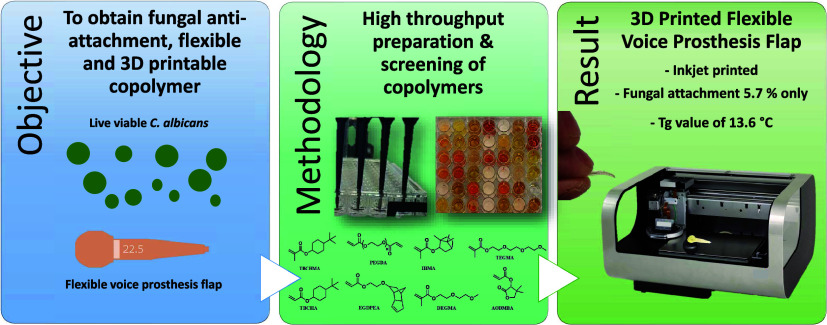

This study reports the development of the first copolymer material that (i) is resistant to fungal attachment and hence biofilm formation, (ii) operates via a nonkilling mechanism, i.e., avoids the use of antifungal actives and the emergence of fungal resistance, (iii) exhibits sufficient elasticity for use in flexible medical devices, and (iv) is suitable for 3D printing (3DP), enabling the production of safer, personalized medical devices. *Candida albicans* (*C. albicans*) can form biofilms on in-dwelling medical devices, leading to potentially fatal fungal infections in the human host. Poly(dimethylsiloxane) (PDMS) is a common material used for the manufacture of medical devices, such as voice prostheses, but it is prone to microbial attachment. Therefore, to deliver a fungal-resistant polymer with key physical properties similar to PDMS (e.g., flexibility), eight homopolymers and 30 subsequent copolymers with varying glass transition temperatures (*T*_g_) and fungal antiattachment properties were synthesized and their materials/processing properties studied. Of the copolymers produced, triethylene glycol methyl ether methacrylate (TEGMA) copolymerized with (r)-α-acryloyloxy-β,β-dimethyl-γ-butyrolactone (AODMBA) at a 40:60 copolymer ratio was found to be the most promising candidate by meeting all of the above criteria. This included demonstrating the capability to successfully undergo 3DP by material jetting, via the printing of a voice prosthesis valve-flap using the selected copolymer.

## 1 Introduction

3D printing (3DP) is a collective term that describes a range of advanced manufacturing techniques for directly fabricating products, many of which adopt a layer-by-layer material deposition strategy. Consequently, these technologies show great potential to revolutionize the manufacture of customizable biomedical devices for use in healthcare.^1−3^ For instance, 3DP-produced heart valves have allowed the development of customized valves that fit specifically to a patient’s specific heart anatomy.^4,5^ However, biomedical devices still face significant operational challenges, such as the surface colonization of biofilm-forming pathogens, which is a key concern for long-term in-dwelling biomedical devices. Every year, fungal infections kill more than 1.5 million people,^6^ with *Candida albicans* being one of the most important of these infectious fungal species. *C. albicans* can cause invasive bloodstream infections, which are most typically presented in immunocompromised patients or patients with medical implants.^7,8^ Furthermore, contaminated medical devices can lead to continued dispersal into the bloodstream, triggering additional serious infection complications and/or malfunctioning of the implant.^9^ Both complications often require invasive remedial surgery to remove/replace infected in-dwelling devices, which can be physically, physiologically, and financially stressful for patients.

Therapy with antifungal drugs can help treat such infections, but they are often detected too late or are ineffectively treated due to a combination of factors, e.g., limited antifungal drugs available on the market, drug side effects, and lack of treatment guidelines for certain device contaminations.^8^ Furthermore, frequent misuse of azole-derived antifungal actives has promoted the spread of antifungal resistance. One study found that *C. albicans* isolated from tuberculosis patients was significantly resistant to six azole-derived drugs.^10^ Additionally, *C. albicans* within biofilms commonly exhibits elevated resistance to antifungal drugs, with the extracellular matrix produced to form the biofilm providing some direct protection. This greatly increases the health risk for the patient and the challenge of administering successful treatment.^11^ Additionally, the incorporation of actives increases the risk of health-related leaching problems, e.g., the potential risk of silver ingestion from the inclusion of nanoparticles or with the potential for organ damage at high antifungal dosages.^12^

The risk of fungal infections is also dependent on the location of a biomedical device. For example, 3DP manufacture of heart valves and voice prostheses not only differ in their function, design, and material requirements to meet the specialized needs of cardiovascular and laryngectomy patients, respectively, but*C. albicans* infections differ at these sites also. Hence, this study focused specifically upon voice prostheses, which are exposed to continuous high humidity, the flow of saliva, and the passage of food, drink, and air. Thus, their use exposes patients to a severe risk of *C. albicans* infection.^13^ All of these can lead to the deposition of fungal cells and the growth of a biofilm.^14,15^ For example, it has been reported that increased carbon dioxide (CO_2_) from exhaled breath may also stimulate the yeast form to switch to filamentous growth, hence promoting mature biofilm formation.^16^ Because of these issues, several strategies have been considered for mitigating the growth of fungi on biomedical devices. One of the most common methods has been to integrate antifungal and antimicrobial actives into the matrix material of biomedical devices. Sionov et al. performed an in vitro study of an ethyl cellulose prosthesis coating embedded with clotrimazole, an active that could inhibit the switch from yeast form to hyphae, thus preventing mature biofilm formation. Microscopic images confirm fungi exposed to clotrimazole have a short hyphal outgrowth.^17^ The use of silver oxide to inhibit *C. albicans* has also been evaluated, where a 7% loading of silver oxide incorporated into silicone rubber of the prosthesis flap valve was shown to extend the mean lifetime of voice prostheses from 36 to 110 days.^18^ However, as defined above, such strategies increase the risk of building microbial resistance and health-related leaching problems. Published data show a direct correlation between the use of antibiotics and resistance and that countries with a higher consumption of antibiotics show higher rates of resistance.^19,20^ Polymers containing charged moieties (i.e., cations) have also been investigated for preventing *C. albicans* biofilm formation on voice box prostheses. A study by de Prijck et al. described how quaternary polydimethylaminoethyl methacrylate (PDMAEMA) and polyethylenimine (PEI) covalently bonded to the surface of a silicone rubber-based voice prosthesis could actively inhibit biofilm growth by up to 92%.^21^

More recently, polymers that inherently resist attachment by bacterial and fungal pathogens, including *C. albicans*, have been discovered through high-throughput microarray screening (HTS) methods.^22−24^ The polymer molecular structure alone produces the resistance, so no actives were needed. For example, Crawford et al. successfully demonstrated the protection of wheat crops from fungal infection in a field trial using an antiattachment acrylate polymer derived from these HTS studies.^25^ This strategy is novel because it allows the surfaces of the devices to be tailored to meet the exact demands of the specific end-use application by developing a coating that is resistant to the specific biological species that are present in that environment. Thus, these coatings have the potential to be exploited in a much broader range of scenarios compared to other potential surface modification methods by changing the comonomer set from which the coating is produced from. By comparison, surface preparations, such as plasma treatment, engineering surface roughness, and/or applying more generic hydrophilic coatings onto the device because they modify the surface in only one “utilitarian” fashion, do not have this potential to have such an expansive operational footprint.

One of the polymers that was identified as a “hit” material during HTS screening, i.e., a polymer that exhibited high levels of resistance to the test fungi, was (r)-α-acryloyloxy-β,β-dimethyl-γ-butyrolactone (AODMBA). However, while this material was shown to demonstrate resistance to fungal attachment by applying these methods, the biological mechanism by which this is delivered is not fully understood. We do know that the polymers achieve bioresistance via a passive mechanism that does not kill the bacteria or fungi that contact the surface. From our experience with bacterial antiattachment materials, developing a definitive understanding of the fungal antiattachment mechanisms will not be straightforward and is probably several years away. Thus, while that is an area of ongoing study, it is outside the scope of this study’s aims.

Our findings to date, gained from applying machine learning methods to our current polymer data sets, suggested that the molecular feature that was most strongly associated with low *C. albicans* attachment was a carbonyl functionality, C(O), whereas the presence of methylene nitrile C(CN) groups was most strongly associated with high attachment.^24^ Meanwhile, spores from a range of fungal species showed low attachment with the most hydrophilic materials, i.e., those that exhibited a water contact angle (WCA) of ≤50°, suggesting that this level of hydrophilicity deterred fungal-spore attachment^25^ However, in the case of (vegetative) cells of *C. albicans*, the lead polymers resisting its attachment were more hydrophobic materials with WCAs of 62–96°. Thus, the ability of these polymers to prevent fungal biofilm formation indicates that hydrophilicity alone is not sufficient to predict fungal attachment to a particular material.^24^

In addition, the assessment of the viscosity of the monomer and its reactivity rate had already been assessed and shown to be suitable for processing by 3DP techniques.^24^ The reaction rate was sufficient because of the acrylate functionality, while the large pendant group was believed to increase the free volume in the sample, thus reducing the viscosity it exhibits. Consequently, the fact that this material exhibited this combination of properties speeds up the possibility for producing personalized devices. Unfortunately, while the AODMBA homopolymer was printable, the end product was found to be too rigid for a soft, flexible device like a voice prosthesis. Therefore, in this study, material flexibility, fungal antiattachment properties, and suitability for 3DP were all considered to design a fungal-resistant material suitable for manufacturing flexible biomedical devices, such as a voice prosthesis flap. Thus, the focus was upon understanding the level of fungal antiattachment behavior and *T*_g_ values exhibited by eight homopolymer candidates to design copolymers that could meet the abovementioned criteria.

## 2 Materials and Methods

### 2.1 Materials

The eight monomers used in this study were triethylene glycol methyl ether methacrylate (TEGMA, 729841, Sigma-Aldrich), di(ethylene glycol) methyl ether methacrylate (DEGMA, 447927, Sigma-Aldrich), poly(ethylene glycol) diacrylate (PEGDA, 437441, Sigma-Aldrich) average *M*_n_ 575, (r)-α-acryloyloxy-β,β-dimethyl-γ-butyrolactone (AODMBA, 376361-5, Sigma-Aldrich), ethylene glycol dicyclopentenyl ether acrylate (EGDPEA, 407968, Sigma-Aldrich), isobornyl methacrylate (IBMA, 392111, Aldrich), 4-*tert*-butylcyclohexyl acrylate (TBCHA, 467545, Sigma-Aldrich), and 4-*tert*-butylcyclohexyl methacrylate (TBCHMA, Nourcryl MC 110, Akzo Nobel). The photoinitiator used was 2,2-dimethoxy-2-phenylacetophenone, DMPA (Sigma-Aldrich), which did not require any purification.

### 2.2 Preparation of Polymers in Polystyrene Tissue Culture Plastic (TCP)-Treated 96-Well Plates

The photoinitiator (PI) was solubilized in the selected monomers using ultrasound for 15 min, where the 5 mL glass bottles containing monomers and the initiator were wrapped with aluminum foil (minimizing exposure to UV light). The bottles were then purged with N_2_ for 5 min after they became homogeneous. The solutions were then transferred into 1.5 mL polypropylene microcentrifuge tubes located within the holder of the liquid handling apparatus (Microlab STARlet, Hamilton Robotics, Inc.). A four-channel pipet liquid handling head equipped with 50 μL pipettes (CO-RE nonfilter tips, Hamilton Robotics, Inc.) was then utilized to perform high-throughput dispensing of monomers into the wells of the polystyrene tissue culture plastic (TCP) 96-well plate. For the preparation of copolymers, the individual amount of each monomer was dispensed into a well and automatically pipet mixed with a five-cycle aspiration process. The TCP filled with monomers was then exposed to a Phoseon Technology 365 nm wavelength UV source at approximately 10 cm away for 5 min. This constant 5 min of exposure resulted in a UV energy input within the range of 3000–3500 mJ/cm^2^ according to the measurement with a UV light meter from OAI Instruments. UV energy measurement involved leaving the UV light meter under the lamp at the desired UV light exposure setting and collecting the UV energy information for the set time. The TCP was then put under vacuum at room temperature for 7 days to remove the residual monomer before it was rinsed with isopropanol (IPA) and sterile distilled water (SDW). Each well was rinsed with 100 μL of IPA and then 100 μL of SDW and later soaked in 100 μL of SDW within the incubator at 37 °C for 48 h. SDW was removed from the wells, and the TCP was then placed under UV light in a biosafety cabinet for 20 min to eliminate potential microbial contamination prior to evaluation of fungal attachment.

### 2.3 Bioassay to Determine the Viability of Mammalian Cells in the Presence of TEGMA and AODMBA Homopolymers

4% w/w DMPA was dissolved in TEGMA and AODMBA under sonication for 15 min in the absence of light. Each solution was then purged with nitrogen for 5 min. 131 uL of each solution was then added to a poly(dimethylsiloxane) (PDMS, Sylgard-184) mold yielding a disc with a surface area of 1.9 cm^2^. Discs were fabricated in quadruplicate. Each disc was then irradiated with a 365 nm lamp for 30 min, after which the PDMS mold was removed to leave a free-standing disc. Samples were then placed under vacuum for 7 days before being washed in sterile water for 2 days at 37 °C. Following this, each disc was washed for 30 min in IPA, allowed to air-dry for 30 min, and then subjected to a 30 min postcure. After postcuring, each disc was washed in ethyl acetate for 1 h, then IPA for 1 h, and then sterile water for 1 h. The water wash was repeated twice for a total of 3 water washes, and discs were left in the final water wash overnight. The sterile water was then removed and replaced with 316.7 μL of culture media containing 10% FBS. Each disc was then incubated in media for 24 h before removal of extract media. L929 mouse fibroblasts (P20) were seeded at a density of 20,000 cells/cm^2^ in the wells of a 96-well plate and incubated for 24 h. After this, the culture media was replaced with extract media from the polymer discs and cells were incubated for a further 24 h. Cell viability was assessed using the PrestoBlue cell viability assay and Live/Dead staining. PrestoBlue cell viability was measured using a Tecan Infinite M200 plate reader, while cell images were acquired using a Nikon plate reading widefield fluorescence microscope. Images were processed using ImageJ and CellProfiler.

### 2.4 Screening of Viscosity, Surface Tension, and *Z* Parameter

This analysis was conducted following a high-throughput method developed to determine the suitability of inks for inkjet printing by Zhou et al.,^26^ which used a four-channel pipet liquid handling apparatus (Microlab STARlet, Hamilton Robotics). For viscosity measurements, 300 μL of monomer(s) was automatically dispensed into the respective wells of a polypropylene tissue-culture-treated plate (PP TCP) (260252, Thermo Scientific), as described above. The PP TCP was placed in a temperature chamber to ensure viscosity was measured at a constant room temperature of 25 °C. Pressure during dispensing was recorded by a pressure sensor within the pipettes, and the viscosity could be calculated using eq 1 (where μ is the dynamic viscosity, *Q* is the volumetric flow rate, *R*_t_ is the radius, ρ is the density, *g* is the gravity, and Θ is the contact angle) by generating a pressure versus time curve
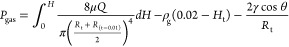
1Surface tension was measured with the assistance of a high-precision balance (WX205SDUV/15, Mettler Toledo) installed into the liquid handling apparatus. 100 μL of monomer was aspirated into a pipet and displaced at a rate of 5 μL/s on the weighing scale. Calculation of surface tension could be determined by eq 2, where γ is the surface tension and *m* is the droplet mass
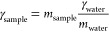
2Fromm’s *Z* parameter, which predicts printability, could be calculated using the dynamic viscosity and surface tension values via eq 3, where μ is the dynamic viscosity, γ is the surface tension, ρ is the density of the ink, and *r* is the nozzle diameter. Mean values of three measurements for both viscosity and surface tension were used to ensure repeatability and estimate/minimize experimental error
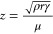
3

### 2.5 Fourier-Transform Infrared (FTIR) Spectroscopy

FTIR spectroscopy was conducted using a Spectrum Two Spectrophotometer (PerkinElmer) equipped with a Universal Attenuated Total Reflection (UATR Diamond/ZnSe). It was used to characterize the degree of conversion (DOC) from the monomer to the polymer, i.e., the level of cure. Spectra were collected within the range of 700–4000 cm^–1^, with three scans obtained to check their repeatability and to minimize experimental error. The spectra were baseline-corrected and normalized to the principal peak in the 1720–1740 cm^–1^ region, which represents the C=O group of acrylate functionality. The C=O group would remain unchanged during the polymerization process. The DOC was then determined via eq 4, by comparing the change in area with those peaks in the 1618–1637 cm^–1^ region, which were defined as C=C. Decreasing the C=C bond level correlates to increasing DOC as the C=C bond converts to C–C bond during polymerization. Thus, tabulated mean values were used in the comparison of DOC, where *A*_C=C_/*A*_C=O_ is the peak area in the polymerized resin and *A*′_C=C_/*A*′_C=O_ is the peak area of the nonpolymerized resin
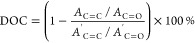
4

### 2.6 Differential Scanning Calorimetry (DSC) Analysis

DSC was conducted on a PerkinElmer DSC8000, and it was used to characterize the *T*_g_ of the polymers and copolymers synthesized. A standard DSC pan from PerkinElmer was used for the measurement, 5–10 mg of samples were placed into the pan, and the lid was secured with a pan crimper press. A reference was prepared with an empty sample pan and placed in the right platinum holder and the sample pan with the actual sample was then placed in the left platinum holder. Both holders were covered with a platinum lid. The DSC profile was programmed to heat from −70 to 200 °C, the materials were heated and cooled twice at a rate of 5 °C/min, and the second heat curve was utilized to analyze *T*_g_ temperature by the extrapolation method.

### 2.7 Evaluation of Fungal Attachment and Polymer Toxicity (XTT Assay)

The XTT tetrazolium salt (2,3-bis(2-methoxy-4-nitro-5-sulfophenyl)-2H-tetrazolium-5-carboxanilide) assay (XTT assay) followed the method reported by Vallières et al.^24^ Wells of TCP were coated with either the test (co)polymer, a positive control (PEGDA), a negative control (AODMBA), or left uncoated as a control. *C. albicans* CAF2-yCherry (provided by R. Wheeler, University of Maine) was grown overnight in 50 mL flasks (inoculated from colonies) containing Yeast Extract-Peptone-Dextrose (YPD) broth at 37 °C with orbital shaking at 150 rpm.^27^ The overnight cultures were washed twice in Roswell Park Memorial Institute (RPMI) 1640 medium and then diluted to 125,000 cells/mL in RPMI medium. Aliquots of 100 μL were transferred to wells of the 96-well plate containing the cured polymers, before static incubation for 2 h at 37 °C. Nonadherent cells were removed by three washes with phosphate buffer saline (PBS). 100 μL of RPMI 1640 was added to the remaining attached cells in each well, and the well plate was incubated statically at 37 °C for 24 h. To measure biofilm metabolic activity after 24 h, the wells were rinsed three times with PBS to remove nonadherent cells, and the reaction was initiated by adding XTT and menadione to fresh RPMI to final concentrations of 210 μg/mL and 4.0 μM (final volume: 200 μL), respectively. After 2 h of static incubation, 100 μL of the reaction solution was transferred to a new 96-well TCP, and formazan absorbance at 490 nm was assessed using a BioTek EL800 microplate spectrophotometer.

Tests for polymer toxicity followed a procedure similar to that for the fungal attachment assay described above. For polymer toxicity, the PBS rinse steps to remove nonadherent cells after 2 h and at 24 h were omitted. The well plate with polymer, RPMI medium, and cells were left in the incubator for 24 h after which the measurement of metabolic activity was measured directly by adding 42 μL of the XTT and menadione mix (with the same concentration as for attachment assays) and 70 μL of fresh RPMI to 88 μL of RPMI remaining in each well. After 2 h, 100 μL of reaction solutions were transferred to a new well plate and absorbance at 490 nm was measured as above. For both “test for polymer toxicity” and “evaluation of fungal attachment”, three replicate tests in independent assay wells (*n* = 3) were evaluated each time to obtain the mean and standard deviation.

### 2.8 Raman Spectroscopy

Raman spectroscopy was performed by using a HORIBA LabRAM HR Raman spectrometer. Spectra were acquired using a 785 nm laser (at ∼20 mW power), a 100× objective, and a 200 μm confocal pinhole. To simultaneously scan a range of Raman shifts, a 300 lines/mm rotatable diffraction grating along a path length of 800 mm was used. Spectra were detected using a Synapse CCD detector (1024 pixels) thermoelectrically cooled to −60 °C. Before spectral collection, the instrument was calibrated using the zero-order line and a standard Si (100) reference band at 520.7 cm^–1^. Similar to FTIR spectroscopy analysis, the application of eq 4 was used to determine the DOC, i.e., by measuring the change in the ratio of peak areas associated with the C=C (∼1640 cm^–1^) and C=O (∼1730 cm^–1^) bonds before and after polymerization.

For single-point spectral measurements, spectra were acquired over the range of 490–1870 cm^–1^ (one spectral window) with 20–60 s integration time and 2–4 accumulations to automatically remove the spikes from cosmic rays and improve the signal-to-noise ratio. For each polymer, three spectra from random locations were acquired under similar conditions. The spectra were then averaged to give a mean.

For multispectral array measurements, spectra were collected (2 s integration time, 1 accumulation, range 490–1870 cm^–1^, 1 spectral window) in 2 μm steps from within a 50 × 50 μm area (676 spectra in total). Spectra were manually despiked, extracted within the range of 500–1400 cm^–1^ and fitted with the component polymer spectra using classic least-squares (CLS) regression analysis within Labspec 6.5 software. False color images were generated by plotting the scores (normalized to a total of 100%) from CLS analysis.

### 2.9 Proton Nuclear Magnetic Resonance (^1^H NMR) Spectroscopy

^1^H NMR was used to evaluate the copolymer ratio within the copolymers and was performed at 25 °C. Samples were prepared as 5–10 mg/mL solutions in deuterated chloroform (CDCl_3_) and pipetted into an NMR sample tube. Spectra were recorded at 400 MHz on a Bruker AV400 instrument at 25 °C and referenced to the residual solvent peak (7.26 ppm for CDCl_3_). Analysis of the spectra was then carried out using Bruker TopSpin 4.1.4 software. Determination of copolymer molar ratios was conducted by referencing the relative integral signals referenced to methoxy group of TEGMA (singlet at 3.39 ppm), three methyl groups of TBCHA (singlet at 1.88 ppm), and methine groups in AODMBA (doublet at 5.35 ppm).

### 2.10 Gel Permeation Chromatography (GPC)

GPC provided information about the polydispersity index (PDI) and molecular weight of the polymers. Samples were dissolved at a concentration of 2 mg/mL in HPLC-grade tetrahydrofuran (THF), allowed to equilibrate for 2 h,and then filtered through a 0.22 μm PTFE syringe filter. The filtered solutions were analyzed by GPC using an LC 1120 HPLC pump eluting in THF at a temperature of 35 °C at a flow rate of 1 mL/min through two Agilent Plgel Mixed-C columns in series to a differential refractive index detector. Calibration was performed using narrow PDI polystyrene standards at a flow rate of 1 mL/min. Analysis was performed offline using ASTRA 6.1 software (Wyatt Technology Corp.).

### 2.11 Ink Preparation

AM inks with homogeneous reagent dispersion and minimal contamination are essential for stable printing performance; hence, the preparation steps include further N_2_ purging and filtering process. The monomers were pipetted into a 5 mL glass vial, which were wrapped in an aluminum foil and equipped with a magnetic stirrer. The appropriate amount of DMPA (either 1 w/v% or 4 w/v%) was then added and the mixture was stirred at 1000 rpm at room temperature for 15–20 min to dissolve the DMPA initiator. Once dissolved, the inks were purged with N_2_ for five min before being filtered through a 5 μm PTFE syringe filter to remove contaminants.

### 2.12 Inkjet Printing

**Figure 1:**
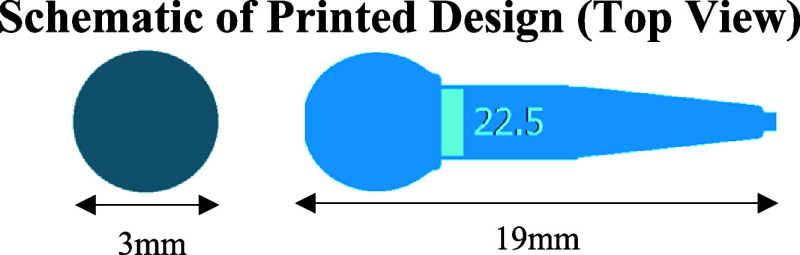
(Left) 3 mm disc-shape pattern used for both DOC and studies using 96-well plate; (Right) 19 mm voice prosthesis flap design.

For the Dimatix materials printer (DMP-2830, Fujifilm, a piezo-based jetting system), the protocol of ink evaluation was developed from the method reported by He et al.^28^ The printer was also enclosed in a nitrogen-purged chamber with oxygen levels kept between 0.25 ± 0.05% during printing to minimize the polymerization-inhibiting effect of O_2_ during the free-radical photopolymerization curing. Inks were filled into 10 pL disposable printheads, Dimatix Materials Cartridge (DMC-11610, Fujifilm) with 16 jet nozzles. Before the start of printing, a new blotting pad was placed in the printer and droplet formation was assessed using the fiducial camera. The in-line UV source came from a 365 nm UV LED unit (800 mW/cm^2^, Printed Electronics Limited, Tamworth, U.K.) and cured the printed ink at an estimated height of 0.5 mm. All of the printed parts were inkjetted on a thin, flexible, and transparent plastic substrate. For a particular ink, the printing parameters were adjusted to obtain stable droplet formation. Cartridge temperature settings were altered in the range from 40 to 55 °C depending on monomer viscosity, with 1–10 kHz firing frequency depending on the curability of the monomer. In order to better compare the different inks and to minimize varying parameters, all inks were printed with 20 μm drop space and the printing voltage was kept constant at 25 V. Inkjet printing was of 70-layer disc-shape with a diameter of 3 mm, such that fungal antiattachment studies and toxicity evaluations could be performed by placing it into a 96-well plate. Similarly, inkjet printing of a 100-layer Voice Prosthesis Flap was achieved by using CAD printing files designed from an actual voice prosthesis flap provided by Atos Medical with the dimensions shown in Figure 1.

### 2.13 Statistical Analysis

Statistical analysis to obtain mean, standard deviation, and p-value was performed using GraphPrism 10.2.3 software. Three replicate tests in independent assay wells (*n* = 3) were evaluated each time. The *p*-values were generated by unpaired *t* tests between polymer candidates and the TCP control. In the figures, *p*-values are denoted as follows: ns, *p* > 0.05; *, *p* ≤ 0.05; **, *p* ≤ 0.01; ***, *p* ≤ 0.001; ****, *p* ≤ 0.0001.

### 2.14 Time-of-Flight Secondary Ion Mass Spectrometry (ToF-SIMS)

A ToF-SIMS IV (IONTOF GmbH) instrument using a 25 keV Bi_3_^+^ primary ion source was used for most of the analysis. Bi_3_^+^ primary ions were used. Analysis for positive and negative spectra was acquired over a 500 μm × 500 μm scan area. Other analyses parameters were a cycle time of 100 μs, one shot/frame/pixel, one frame/patch, and 20 scans per analysis. As the samples were of a nonconductive nature, charge compensation in the form of a low-energy (20 eV) electron flood gun was applied. Images and spectra were acquired using SurfaceLab 6 and analyzed using SurfaceLab 7.1 software.

## 3 Results and Discussion

**Scheme 1:**
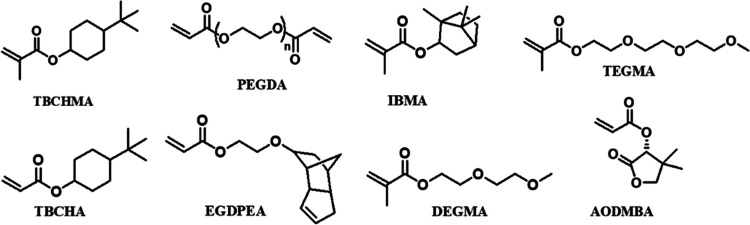
Molecular Structures of Eight Chosen Monomers

Eight candidate monomers were selected by data-mining the results of a previous study that screened 281 methacrylate and acrylate polymers for their fungal antiattachment behavior.^24^ These eight monomers were triethylene glycol methyl ether methacrylate (TEGMA), di(ethylene glycol) methyl ether methacrylate (DEGMA), poly(ethylene glycol) diacrylate (PEGDA), (r)-α-acryloyloxy-β,β-dimethyl-γ-butyrolactone (AODMBA), ethylene glycol dicyclopentenyl ether acrylate (EGDPEA), isobornyl methacrylate (IBMA), 4-*tert*-butylcyclohexyl acrylate (TBCHA), and 4-*tert*-butylcyclohexyl methacrylate (TBCHMA); see Scheme 1 for their molecular structures.

PEGDA was selected as a known positive control for high *C. albicans* attachment, while IBMA and EGDPEA were included as they have reported bacterial antiattachment properties,^29,30^ and so gave the potential of jointly preventing both fungal and bacterial attachment.

**Figure 2:**
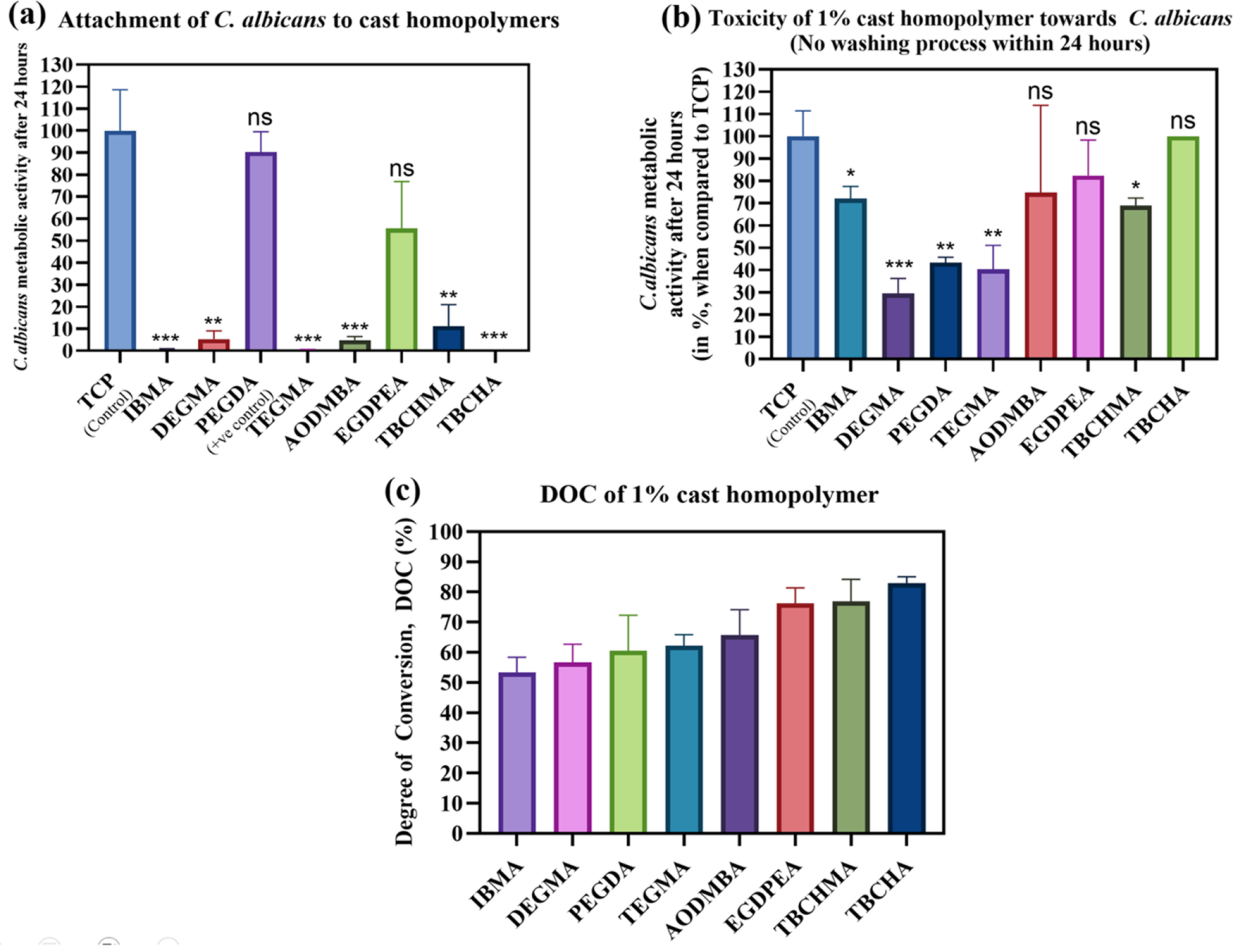
(a) Homopolymer resistance to *C. albicans* attachment. Error bars indicate SD from three replicate measurements, i.e., *n* = 3, whereas symbols above the bars indicate statistical significance compared to the TCP control, as defined in Section 2.13; ns, not significant. (b) Potential toxicity toward *C. albicans* of the homopolymers (*n* = 3). The difference in these assays compared to (a) was the absence of a washing step; both were compared against the results of adherence to TCP. Error bars and tests of significance are as for panel (a). (c) Degree of conversion (DOC) of homopolymer with 1 w/v% (PI), assessed with ATR-FTIR (*n* = 3).

These monomers were homopolymerized in wells of a TCP 96-well plate using 1 w/v% 2,2-dimethoxy-2-phenylacetophenone (DMPA) photoinitiator (PI) as the radical source, and the resultant polymers were subjected to bioassay analysis. Vallières et al. reported that AODMBA, TBCHA, TBCHMA, TEGMA, and DEGMA all exhibited fungal attachment levels of <25% that of the TCP well control, which represents the negative control, i.e., a well without any coating.^24^ Therefore, this was used as the definition of attachment-resistant behavior in this study. The results from the attachment assays are displayed in Figure 2a. The data showed that all tested materials, except for EGDPEA and PEGDA (positive control), were able to resist *C. albicans* colonization, i.e., exhibited <25% *C. albicans* attachment and *p* < 0.05 compared to the TCP control. Values were 0% for negative controls not inoculated with the yeast.

Meanwhile, toxicity evaluation showed that three of the five nonpoly(ethylene oxide)-containing materials (i.e., AODMBA, EGDPEA, and TBCHA) had no significant toxic effect to *C. albicans*, while TBCHMA and IBMA still achieved >65% retention of *C. albicans* metabolic activity after interaction with the polymers for 24 h when compared to the TCP material (Figure 2b). This corroborated that the extremely low levels of *C. albicans* attachment to these polymers (excluding EGDPEA) were not due to accidental toxicity effects associated with the test materials. These five polymers were also found to retain a glassy physical form in the presence of a bioassay medium.

In contrast, monomethacrylate polymers with only poly(ethylene oxide) pendant groups, i.e., DEGMA and TEGMA, were observed to exhibit “swelling”. This phenomenon was attributed to a combination of their low *T*_g_ (−36 and −53 °C, respectively; see Figures S1 and S2), their hydrophilic nature, and a lower level of cure because methacrylate monomers are known to cure slower than acrylates.^31^ The latter is evidenced in Figure 2c, which shows that the experimentally achieved degree of conversion (DOC) was only 56.6% for DEGMA and 62.1% for TEGMA. This observation was also linked to their greater toxicity due to a higher level of free monomer.

All eight monomers were also included in an inkjet printing trial and were formulated into printing “inks” containing 1% PI, to keep the quantity of the PI, a potentially toxic material, to a minimum. However, only AODMBA and PEGDA were found to form stable printed layers at this PI level. Thus, the PI concentration was increased to 4 w/v%, a level that had been previously reported to improve the curing of methacrylate inks.^32^ Visual inspection of the 4% (w/v) PI loading prints confirmed that these prints did exhibit improved cure levels (see Table 1 and Figure S3).

**Table 1:** Summary of the Homopolymer’s *T*_g_, Cast-Cured Physical Form, Jet-Ability, and Curing with 1 and 4 w/v% PI^a^

monomer	*T*_g_ (°C)	physical form in cast UV-cured	jettable	optimum jetting freq (kHz)	3DP with 1% w/v DMPA	3DP with 4% w/v DMPA
PEGDA	–24	glassy	Y	5	Y	Y
AODMBA	86	glassy	Y	10	Y	Y
TBCHA	121	glassy	Y	5	N	Y
TBCHMA	159	glassy	Y	1	N	Y
IBMA	88	glassy	Y	NA^b^	N	gel-like
TEGMA	–53	rubbery	Y	1	N	gel-like
EGDPEA	23	glassy	Y	1	N	Y
DEGMA	–36	rubbery	Y	1	N	gel-like

a Polymer cast curability assessed by visual observation only.

b IBMA was not printable even at 1 kHz and hence no optimum jetting frequency identified for IBMA.

During printing, the UV exposure time was optimized for each monomer. It was found to be possible to cure the AODMBA 4 w/v% PI ink with the lowest level of UV light exposure (Dimatix frequency setting of 10 kHz). By comparison, the TBCHA 4 w/v% ink required a longer UV exposure (5 kHz). Meanwhile, with TBCHMA, EGDPEA, TEGMA, and DEGMA, some form of curing was achievable only with the longest UV light exposure time at a frequency of 1 kHz. Additionally, while the increase in PI loading improved the level of cure obtained with TEGMA, DEGMA, and IBMA inks, their physical appearance was soft and gel-like after printing. For TEGMA and DEGMA, this was attributed to their subroom temperature (RT) *T*_g_ values. Meanwhile, it was proposed that the 3DP IBMA had achieved a suboptimal monomer-to-polymer conversion due to the bulky isobornyl group causing steric hindrance around the polymerization center.^33−35^

The ultimate plan for these materials is to utilize them to manufacture a prosthetic voice box using additive-manufacturing/3D printing techniques. Thus, as these materials will be in contact with the tissue of the larynx, the toxicity of the favored polymers was assessed to ensure that there would not be negative therapeutic consequences from the implanting of a medical device made from these materials. Thus, a bioassay to determine the viability of mammalian cells (L929 mouse fibroblasts) cultured in the presence of the extract media of homopolymers TEGMA and AODMBA was conducted, and this data is shown in Figure S4. This data will cover any combination of these monomers subsequently used in this and follow-on trials. This assay demonstrated that there was a good viability of the cells when in contact with the extract media of these materials (>75% live cells). Thus, it was defined that these materials are nontoxic to mammalian cells and so appropriate to use in implanted medical devices.

### 3.1 Copolymer Optimization Study

The selected monomers were subjected to DSC analysis (see Table 1 and Figures S1, S2, and S5–S10). The sub-RT TEGMA and DEGMA homopolymers had previously been evaluated to be very promising fungal antiattachment candidates. However, they were concluded to have limited potential as fungal antiattachment polymers for biomedical devices.^24^ This is because these polymers could exhibit creep behavior when in use, leading to a loss of structural fidelity or even failure of the final device. The voice box flap must be flexible to be able to move relative to the main “body” of the prosthesis to allow the egress of gases but prevent the ingress of liquids. In a prior study investigating a medical device application that requires similar levels of flexibility, i.e., coating silicone urinary catheters with a bacteria-resistant polymer, a coating *T*_g_ between −5 and 15 °C was reported to allow the coating to flex adequately in-use.^36^ Therefore, this was used as a target *T*_g_ range in this study. Therefore, copolymers were synthesized by combining TEGMA and DEGMA with comonomers that produce higher *T*_g_ homopolymers (EGDPEA, IBMA, AODMBA, TBCHA, TBCCHMA) (DSC data as per Figures S6–S9) to increase the *T*_g_ of the final material while retaining the fungal-resistant behavior. The expected *T*_g_ for 30 specific copolymer feed ratios, i.e., 25:75, 50:50, and 75:25 v/v% ratios were calculated using the Flory–Fox equation (eq 5), where *w*_1_ is the mass fraction of the first monomer and *w*_2_ is the mass fraction of the second^37^
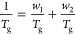
5From this list, the four copolymers detailed in Table 2 were noted to have predicted *T*_g_ values that were in the target range. Thus, cast samples of these copolymers were prepared using both 1 and 4 w/v% PI loadings, and *C. albicans* attachment was measured.

**Table 2:** Summary of the Predicted Copolymer’s *T*_g_ by Flory–Fox Equation and *C. albicans* Attachment Data Measured on Cast Samples Cured Using 1 and 4 w/v% PI

copolymer	*T*_g_^a^ (°C)	*C. albicans* attachment^b^ to cast copolymers with 1% w/v DMPA (%)	*C. albicans* attachment^b^ to cast copolymers with 4% w/v DMPA (%)
TEGMA:AODMBA_50:50_	0.0	1.3 ± 1.2****	2.3 ± 4.0****
TEGMA:TBCHA_50:50_	11.5	2.5 ± 2.3****	5.0 ± 4.4****
TEGMA:IBMA_50:50_	0.5	17.8 ± 11.2***	13.8 ± 2.1****
DEGMA:AODMBA_50:50_	12.5	0.79 ± 1.4****	12.7 ± 14.6***

a Predicted by Flory–Fox equation.

b Values shown are means **±** SD (*n* = 3). Asterisks indicate statistical significance versus controls as defined in Section 2.13.

The results showed that all of the samples demonstrated <25% attachment at both initiator levels (Table 2). However, before direct inkjet printing trials with these copolymers were conducted, cast samples of TEGMA:AODMBA and TEGMA:TBCHA copolymers were prepared to confirm that the copolymerization had taken place, and the ratios of the copolymers closely aligned with the feed ratio employed. For both, copolymer ratios of 75:25, 50:50, and 25:75 were prepared and analyzed to determine the trends in (a) the DOC achieved, (b) copolymer ratio and relative spatial distribution achieved via Raman mapping, and (c) the fungal attachment when the casts were prepared with 1% PI, as this will be the worst-case scenario. The DOC was assessed via two methods (a) FTIR and (b) Raman spectroscopy, and these data are shown in Table 3.

**Table 3:** Material Property Data for Copolymers^a^

polymer	DOC (FTIR) (*n* = 1) (%)	DOC (Raman) (*n* = 3) (%)	TEGMA CLS score (%)	AODMBA CLS score (%)	TBCHA CLS score (%)	fungal^b^ antiattachment (%)
TEGMA_100_	68.5	71.3 ± 0.5				
TBCHA_100_	100.0	99.5 ± 0.0				
AODMBA_100_	80.2	89.0 ± 0.1				
TEGMA:AODMBA_75:25_	97.1	100.0 ± 0.0	73.1 ± 0.6	26.9 ± 0.6		2.6 ± 1.6****
TEGMA:AODMBA_50:50_	98.0	100.0 ± 0.0	50.6 ± 0.6	49.4 ± 0.6		2.3 ± 4.0****
TEGMA:AODMBA_25:75_	100.0	100.0 ± 0.0	24.9 ± 0.6	75.1 ± 0.6		63.6 ± 10.4**
TEGMA:TBCHA_75:25_	85.8	66.5 ± 0.1	77.0 ± 0.5		23.0 ± 0.5	9.5 ± 8.5***
TEGMA:TBCHA_50:50_	100.0	98.2 ± 0.2	47.1 ± 0.5		52.9 ± 0.5	5.0 ± 4.4****
TEGMA:TBCHA_25:75_	100.0	100.0 ± 0.0	20.9 ± 0.8		79.1 ± 0.8	29.6 ± 21.2**

a (a) Degree of conversions (DOC) evaluated using FTIR and Raman spectroscopy (*n* = 3) (± figures represent the standard deviation), (b) polymer ratios obtained by classic least squares (CLS) analysis of Raman multispectral data sets for the selected copolymers, and (c) fungal antiattachment (%) on polymers with 4% DMPA assessed with *C. albicans* metabolic activity after 24 h.

b Values shown are means ± SD (*n* = 3). Asterisks indicate statistical significance versus controls as defined in Section 2.13.

DOC values determined by the two spectroscopy methods were generally in good agreement for all copolymer materials assessed (Table 3). TEGMA:TBCHA_75:25_ was an exception, where the DOC values obtained from FTIR and Raman spectra showed a difference of approximately 20%. This disparity was attributed to the steric bulk of the pendent group reducing the efficiency of the copolymerization reaction. This data also showed that increased levels of an acrylate monomer also increased DOC, when compared to the homopolymers, with all of the 50:50 and 25:75 materials reaching close to 100% DOC (Table 3). Furthermore, Raman imaging and subsequent classic least-squares (CLS) regression analysis of the multispectral data sets provided additional information on the quantities and distributions of the respective polymers within the copolymers (see Table 3 and Figures S11–S16). The false color images and consolidated CLS scores for all copolymer variants showed that the (a) copolymer ratios were close to the reagent feed ratios (Table 3) and (b) polymers were homogeneously spread throughout both the TEGMA:AODMBA and TEGMA:TBCHA copolymers; i.e., there was no evidence of heterogeneity in any of the copolymer samples.

**Figure 3:**
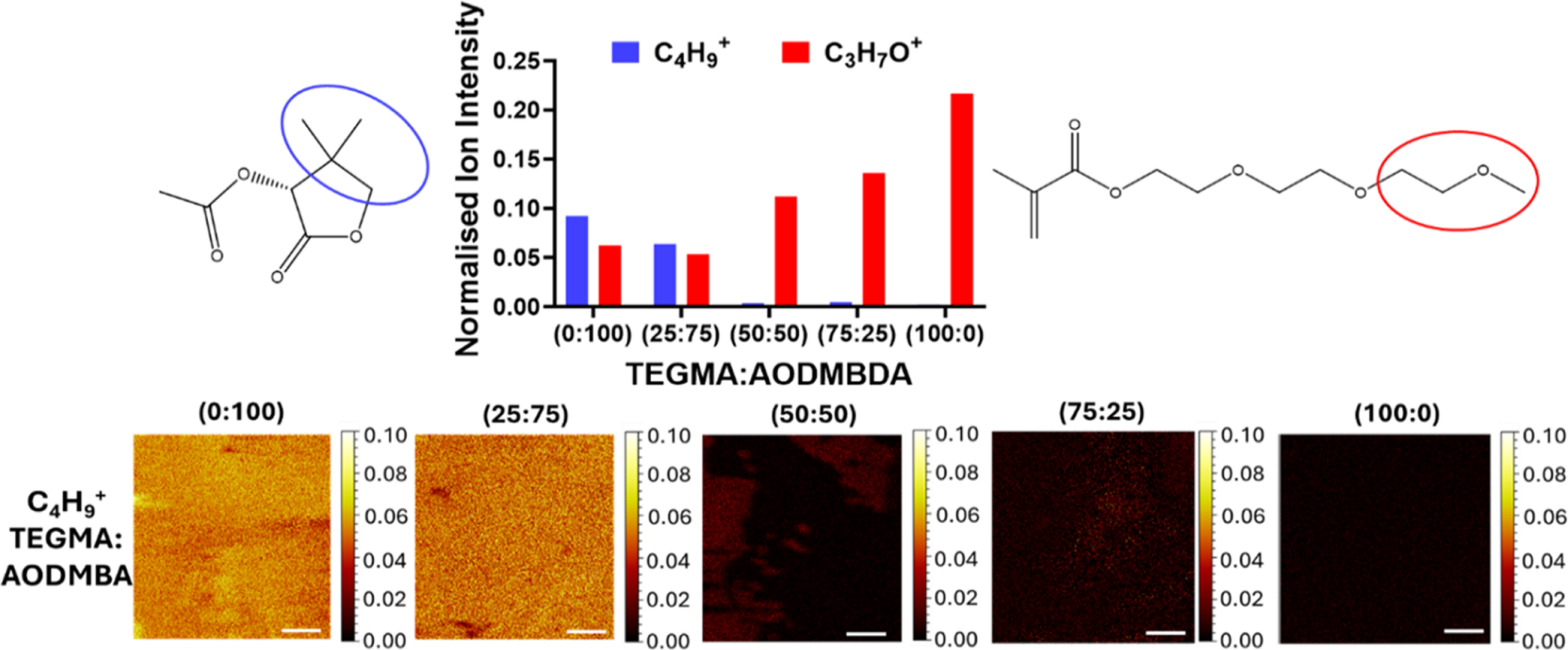
ToF-SIMS data of the AODMBA:TEGMA series showing assignment of C_4_H_9_^+^ (blue) to AODMBA and C_4_H_7_O^+^ (red) to TEGMA. Chemical map images show distribution of the AODMBA C_4_H_9_^+^ ion over the samples. Scale bar equivalent to 100 μm.

Additionally, the attachment data exhibited the expected trend with only the materials with at least 50% TEGMA demonstrating attachment levels <25%. A copolymer series of TEGMA:AODMBA (0:100, 25:75, 50:50, 75:25, and 100:0) was synthesized and analyzed using time-of-flight secondary ion mass spectrometry (ToF-SIMS) to observe trends in surface chemistry, as shown in Figure 3.

**Figure 4:**
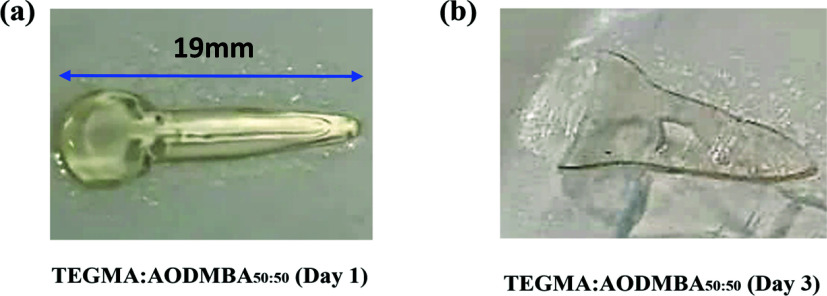
(a) Image of successful printing with TEGMA:AODMBA_50:50_ into a voice prosthesis tongue. (b) Image showing that the printed part exhibited loss of structural fidelity due to creep after 3 days of storage at room temperature.

A unique ion for AODMBA was identified (C_4_H_9_^+^) where this ion is present in AODMBA but not in TEGMA. Figure 3 shows that once the copolymer ratio reaches 50:50, TEGMA starts to dominate the surface, as no C_4_H_9_^+^ ion is present on the surface. This suggests that the antifungal behavior benefits from having TEGMA present on the material surface, as above 50% TEGMA was shown to keep attachment levels less than 25%. The associated positive mass spectra (50–100 *m*/*z*) and the overlay of the C_4_H_9_^+^ peak can be found in Figures S17 and S18. Thus, since the 50:50 copolymers were the variants that exhibited both the target *T*_g_ and fungal resistance needed for the end-use application, these were the variants taken into 3DP trials. Initially, the viscosity and surface tension of these inks were then assessed for their suitability for printing. This was achieved by jointly screening these properties using a high-throughput liquid handling apparatus. These data (see Table S1) were then used to calculate Fromm’s *Z* parameter using eq 6,^26^ where *r* is the printer nozzle diameter, ρ is the comonomer density, γ is the surface tension and μ the dynamic viscosity
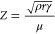
6The calculated *Z* parameters for TEGMA:AODMBA_50:50_ and TEGMA:TBCHA_50:50_ materials were 3.1 and 4.7, respectively (see Table S1), which fall within the ideal range specification of 1–10 for good droplet formation.^38^ Subsequently, a preliminary inkjet print trial with the copolymers was performed to test the printing performance of these comonomer inks. This trial showed that, even at PI loading of 4%, the TEGMA:TBCHA_50:50_ was unable to form solid layers, even after fine-tuning the printing parameters to have the longest UV light exposure at a frequency of 1 kHz. Hence, this copolymer was not assessed further. Meanwhile, TEGMA:AODMBA_50:50_ exhibited good layer formation with the same frequency of 1 kHz (Figure 4a), but the print had a poor printing resolution and also showed evidence of creep after 3 days of storage, as seen in Figure 4b.

It was proposed that the observed level of creep/lack of fidelity in this 3DP application may be attributed to the fact that the material was not applied as a coating, as seen in previous studies.^36^ In those studies, the material was applied to a preformed medical device, the matrix materials of which imparted structural consistency. Therefore, it was concluded that either (a) the *T*_g_ of 0 °C was still too low in this case or (b) the degree of curing in the printed system was lower compared to that observed in the cast samples. Meanwhile, analysis of the TEGMA:AODMBA_25:75_ copolymer had already been shown to have a *T*_g_ that was too high for the application (Table 1). Consequently, the material properties of cast samples TEGMA:AODMBA copolymers containing additional comonomer ratios of 35:65, 45:55 were investigated to determine if increasing the level of higher *T*_g_ acrylate monomer (AODMBA) within the copolymer was sufficient to prevent creep in 3DP parts. Furthermore, fungal attachment assays were performed for each variant to relate the changes in the copolymer ratio to the level of resistance observed. This data are summarized in Table 4 and include copolymer analysis from GPC (Figures S19–S21), ^1^H NMR spectroscopy (Figures S22–S24), and DSC (Figure S25).

**Table 4:** Copolymer Data for TEGMA:AODMBA Materials with Targeted vol:vol Comonomer Feed Ratios of 35:65, 40:60, and 50:50 Evaluated for Their (a) ^1^H NMR Spectroscopy Copolymer Ratio, (b) Number-Average Molecular Weight (*M*_n_), (c) Polydispersity Index (PDI), (e) DOC Values, and (f) Fungal Attachment (*n* = 3) (± Symbol Representing the Standard Deviation)^a^

			*T*_g_^d^			
copolymer^b^ ratio achieved (%)	*M*_n_^c^ (g mol^–1^)	*Đ*^c^	calc (°C)	DSC (°C)	DOC^e^ (%)	fungal attachment^f^ (%)
38.1:61.9	58,000	3.39	21.4	22.7	87.2	59.0 ± 10.8**
41.5:58.5	59,000	3.11	14.0	13.6	94.6	5.7 ± 3.1****
51.2:48.8	50,500	3.81	0.1	–2.0	90.4	1.8 ± 0.4****

a Values shown are means ± SD (*n* = 3). Asterisks indicate statistical significance versus controls as defined in Section 2.13.

b Defined via NMR analysis.

c Defined via GPC analysis.

d Defined by DSC analysis.

e Defined by FTIR spectroscopy.

f Defined via XTT assay and compared to TCP.

Changing the feed ratio of TEGMA:AODMBA from 50:50 to 35:65 in the cast samples led to a significant order of magnitude increase in fungal attachment when compared to that of PEGDA (negative control). In contrast, the 40:60 feed ratio was observed to retain a low level of fungal attachment. Meanwhile, the molecular weight of polymers was also an important consideration as it can have a significant impact on the final mechanical properties. All three copolymers had comparable, high number-average molecular weight (*M*_n_) of at least 50,000 g mol^–1^ (see Table 4). The resulting PDI values were also similar and relatively typical of this type of initiator-controlled bulk polymerization process, leading to high conversion. GPC results revealed the presence of several smaller peaks, potentially attributed to the existence of oligomers (refer to Figures S17–S19). These shorter-chain polymers may form due to poor diffusion caused by increased viscosity during the bulk UV polymerization process. NMR spectroscopy data also confirmed the Raman mapping results, showing that the copolymer ratios were close to the feed ratios applied in the copolymer preparation. Furthermore, vinyl peaks are absent in all NMR spectra (Figures S22–S24) confirming that much of the monomer had been converted into copolymer (see Table 4).

**Figure 5:**
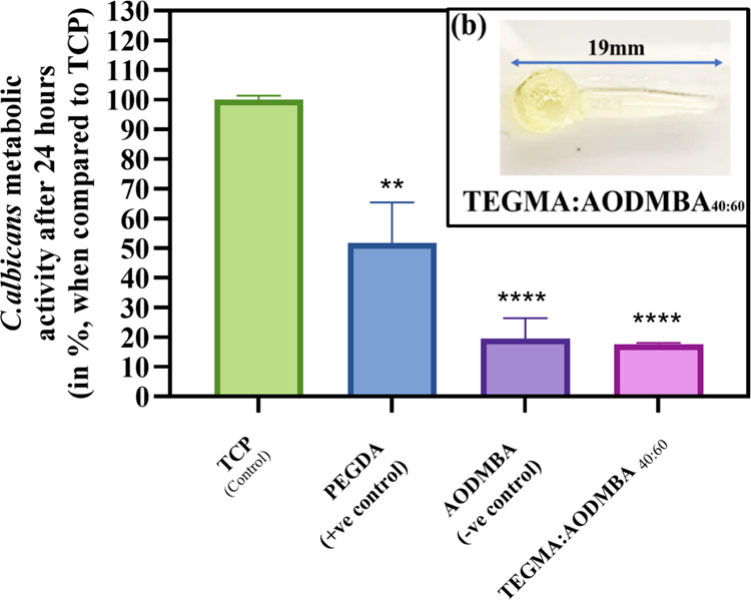
Main body shows that Inkjet-printed TEGMA:AODMBA_40:60_ was printed into a 3.5 mm × 1.5 mm disc shape and placed into a 96-well plate to evaluate the *C. albicans* attachment, compared against PEGDA (+ve control) and AODMBA (-ve control) Error bars indicate SD from three replicate measurements, i.e., *n* = 3, whereas symbols above bars indicate statistical significance compared to the TCP control, as defined in Section 2.13; ns, not significant. (b) TEGMA:AODMBA_40:60_ was printed into a voice prosthesis flap.

Consequently, TEGMA:AODMBA_40:60_ was selected to investigate if the new copolymer ratio would mitigate the problem of the polymer creeping/lack of fidelity observed with TEGMA:AODMBA_50:50_, as the *T*_g_ value of TEGMA:AODMBA_40:60_ was close to the top end of the target range (for DSC spectra see Figure S25) and it had delivered low levels of attachment. Then, 3 mm diameter × 1.5 mm thickness discs of TEGMA:AODMBA_40:60_ were inkjet-printed (70 layers using a frequency of 1 kHz) to obtain samples that could be fitted into 96-well TCP to evaluate its fungal antiattachment property in the printed form. These printed discs were incubated with *C. albicans* alongside printed PEGDA and AODMBA homopolymers to act as positive and negative controls, respectively. The discs printed with TEGMA:AODMBA_40:60_ copolymers successfully gave reproducible fungal antiattachment levels of <25%, as shown in Figure 5 main body.

The printed voice prosthesis consisting of a TEGMA:AODMBA_40:60_ copolymer also produced an article that exhibited better definition and no creep behavior as can be seen in Figure 5b. It was concluded that this supported the hypothesis that *T*_g_ of these polymers is an important feature of the materials because this copolymer exhibited a *T*_g_ that fell into the target range.

Thus, this material has been shown to meet both criteria considered for this work, i.e., to be both flexible and inhibit fungal attachment. Furthermore, it was shown to be possible to 3DP a viable voice prosthesis flap with the comonomer inks of TEGMA:AODMBA_40:60_. This meant that it could be successfully handled (manually flexed) and did not exhibit creep. The results support the use of copolymerization approaches for successful selection of TEGMA:AODMBA_40:60_ among other potential materials that, alongside other appropriate properties including flexibility, remained resistant to fungal attachment after printing.

## 4 Conclusions

This manuscript reports the first successful additive-manufacturing-based production of a fungal-resistant, flexible, voice prosthesis valve-flap that prevents fungal attachment without killing the biological species that contact the surface. Material property, bioassay, printability screening, and calculation of *T*_g_ for 30 potential copolymers were assessed. This analysis concluded that TEGMA:AODMBA_50:50_, TEGMA:TBCHA50:50, TEGMA:IBMA_50:50_, and DEGMA:AODMBA_50:50_ when cured in a casting process with 4 w/v% DMPA PI exhibited both the target fungal antiattachment level of <25% of a positive control’s attachment levels, and a *T*_g_ that would give an appropriate level of flexibility for successful operational performance in the chosen prosthesis. TEGMA:AODMBA_50:50_ and TEGMA:AODMBA_40:60_ copolymers were also found to be suitable for inkjet printing, with the printed copolymer of TEGMA:AODMBA_40:60_ retaining both fungal antiattachment (<25%) and the fidelity of their structural dimensions/material properties. Thus, this work demonstrated a novel approach of preparing flexible 3DP formulations for biomedical devices, such as voice prosthesis components.

## Supporting Information Available

DSC analysis data collected to determine the glass transition temperatures of homopolymers and the TEGMA:AODMBA_40:60_ copolymer used for 3D printing; ^1^H NMR spectra and GPC chromatograms of TEGMA:AODMBA_35:65_, TEGMA:AODMBA_40:60_, and TEGMA:AODMBA_50:50_ copolymers; pictorial Images taken by fiducial camera of Dimatix DMP-2830 showing the left top quarter of 3 mm circular discs printed as a two-layer print with PEGDA, TBCHA, TBCHMA, and AODMBA homopolymers; false color images obtained by Raman spectroscopy analysis of the 75:25, 50:50, and 25:75 ratios of TEGMA:AODMBA and TEGMA:TBCHA copolymers; room-temperature viscosity and surface tension data for TEGMA:AODMBA_50:50_ and TEGMA:TBCHA_50:50_ copolymers synthesized using 4% w/v DMPA initiator; and bioassay data used to determine the viability of mammalian cells in the presence of TEGMA and AODMBA homopolymers (DOCX)

## References

[ref1] Singh S.; Ramakrishna S. Biomedical Applications of Additive Manufacturing: Present and Future. Curr. Opin. Biomed. Eng. 2017, 2, 105–115. 10.1016/j.cobme.2017.05.006.

[ref2] Beg S.; Almalki W. H.; Malik A.; Farhan M.; Aatif M.; Rahman Z.; Alruwaili N. K.; Alrobaian M.; Tarique M.; Rahman M. 3D Printing for Drug Delivery and Biomedical Applications. Drug Discovery Today 2020, 25 (9), 1668–1681. 10.1016/j.drudis.2020.07.007. 32687871

[ref3] Kumar R.; Kumar M.; Chohan J. S. The Role of Additive Manufacturing for Biomedical Applications: A Critical Review. J. Manuf. Processes 2021, 64, 828–850. 10.1016/j.jmapro.2021.02.022.

[ref4] Bhandari S.; Yadav V.; Ishaq A.; Sanipini S.; Ekhator C.; Khleif R.; Beheshtaein A.; Jhajj L. K.; Khan A. W.; Al Khalifa A.; Naseem M. A.; Bellegarde S. B.; Nadeem M. A. Trends and Challenges in the Development of 3D-Printed Heart Valves and Other Cardiac Implants: A Review of Current Advances. Cureus 2023, 15 (8), e43204 10.7759/cureus.43204. 37565179 PMC10411854

[ref5] Ginty O.; Moore J.; Peters T.; Bainbridge D. Modeling Patient-Specific Deformable Mitral Valves. J. Cardiothorac. Vasc. Anesth. 2018, 32 (3), 1368–1373. 10.1053/j.jvca.2017.09.005. 29221976

[ref6] Bongomin F.; Gago S.; Oladele R. O.; Denning D. W. Global and Multi-National Prevalence of Fungal Diseases—Estimate Precision. J. Fungi 2017, 3 (4), 57 10.3390/jof3040057. PMC5753159 29371573

[ref7] Shoham S. Invasive Candidiasis in Patients with Implants. Curr. Fungal Infect. Rep. 2011, 5 (1), 12–17. 10.1007/s12281-010-0040-8.

[ref8] Nobile C. J.; Johnson A. D. *Candida albicans* Biofilm and Human Disease. Annu. Rev. Microbiol. 2015, 69, 71–92. 10.1146/annurev-micro-091014-104330. 26488273 PMC4930275

[ref9] Kojic E. M.; Darouiche R. O. Candida Infections of Medical Devices. Clin. Microbiol. Rev. 2004, 17 (2), 255–267. 10.1128/CMR.17.2.255-267.2004. 15084500 PMC387407

[ref10] Dhasarathan P.; AlSalhi M. S.; Devanesan S.; Subbiah J.; Ranjitsingh A. J. A.; Binsalah M.; Alfuraydi A. A. Drug Resistance in Candida Albicans Isolates and Related Changes in the Structural Domain of Mdr1 Protein. J. Infect. Public Health 2021, 14 (12), 1848–1853. 10.1016/j.jiph.2021.11.002. 34794907

[ref11] Kaur J.; Nobile C. J. Antifungal Drug-Resistance Mechanisms in *Candida* Biofilms. Curr. Opin. Microbiol. 2023, 71, 102237 10.1016/j.mib.2022.102237. 36436326 PMC11569868

[ref12] Zhou Z.-X.; Yin X.-D.; Zhang Y.; Shao Q.-H.; Mao X.-Y.; Hu W.-J.; Shen Y.-L.; Zhao B.; Li Z.-L. Antifungal Drugs and Drug-Induced Liver Injury: A Real-World Study Leveraging the FDA Adverse Event Reporting System Database. Front. Pharmacol. 2022, 13, 891336 10.3389/fphar.2022.891336. 35571077 PMC9098189

[ref13] Talpaert M. J.; Balfour A.; Stevens S.; Baker M.; Muhlschlegel F. A.; Gourlay C. W. *Candida* Biofilm Formation on Voice Prostheses. J. Med. Microbiol. 2015, 64 (3), 199–208. 10.1099/jmm.0.078717-0. 25106862

[ref14] Seneviratne C. J.; Jin L.; Samaranayake L. P. Biofilm Lifestyle of Candida: A Mini Review. Oral Dis. 2008, 14 (7), 582–590. 10.1111/j.1601-0825.2007.01424.x. 19076549

[ref15] Cavalheiro M.; Teixeira M. C. *Candida* Biofilms: Threats, Challenges, and Promising Strategies. Front. Med. 2018, 5, 28 10.3389/fmed.2018.00028. PMC5816785 29487851

[ref16] Pentland D. R.; Davis J.; Mühlschlegel F. A.; Gourlay C. W. CO_2_ Enhances the Formation, Nutrient Scavenging and Drug Resistance Properties of *C. albicans* Biofilms. npj Biofilms Microbiomes 2021, 7 (1), 67 10.1038/s41522-021-00238-z. 34385462 PMC8361082

[ref17] Sionov R. V.; Gati I.; Kirmayer D.; Friedman M.; Steinberg D.; Gross M. Voice Prosthesis Coated with Sustained Release Varnish Containing Clotrimazole Shows Long-Term Protection against *Candida albicans*: An in Vitro Study. Molecules 2021, 26 (17), 5395 10.3390/molecules26175395. 34500827 PMC8434179

[ref18] Kress P.; Schäfer P.; Schwerdtfeger F. P. Clinical Use of a Voice Prosthesis with a Flap Valve Containing Silver Oxide (Blom-Singer Advantage), Biofilm Formation, in-Situ Lifetime and Indication. Laryngorhinootologie 2006, 85 (12), 893–896. 10.1055/s-2006-925292. 16612757

[ref19] Riedel S.; Beekmann S. E.; Heilmann K. P.; Richter S. S.; Garcia-de-Lomas J.; Ferech M.; Goosens H.; Doern G. V. Antimicrobial Use in Europe and Antimicrobial Resistance in *Streptococcus pneumoniae*. Eur. J. Clin. Microbiol. Infect. Dis. 2007, 26 (7), 485 10.1007/s10096-007-0321-5. 17551759

[ref20] Goossens H.; Ferech M.; Vander Stichele R.; Elseviers M. Outpatient Antibiotic Use in Europe and Association with Resistance: A Cross-National Database Study. Lancet 2005, 365 (9459), 579–587. 10.1016/S0140-6736(05)17907-0. 15708101

[ref21] de Prijck K.; de Smet N.; Coenye T.; Schacht E.; Nelis H. J. Prevention of *Candida albicans* Biofilm Formation by Covalently Bound Dimethylaminoethylmethacrylate and Polyethylenimine. Mycopathologia 2010, 170 (4), 213–221. 10.1007/s11046-010-9316-3. 20458631

[ref22] Hook A. L.; Anderson D. G.; Langer R.; Williams P.; Davies M. C.; Alexander M. R. High Throughput Methods Applied in Biomaterial Development and Discovery. Biomaterials 2010, 31 (2), 187–198. 10.1016/j.biomaterials.2009.09.037. 19815273

[ref23] Hook A. L.; Chang C. Y.; Yang J.; Luckett J.; Cockayne A.; Atkinson S.; Mei Y.; Bayston R.; Irvine D. J.; Langer R.; Anderson D. G.; Williams P.; Davies M. C.; Alexander M. R. Combinatorial Discovery of Polymers Resistant to Bacterial Attachment. Nat. Biotechnol. 2012, 30 (9), 868–875. 10.1038/nbt.2316. 22885723 PMC3796337

[ref24] Vallières C.; Hook A. L.; He Y.; Crucitti V. C.; Figueredo G.; Davies C. R.; Burroughs L.; Winkler D. A.; Winkler D. A.; Winkler D. A.; Winkler D. A.; Wildman R. D.; Irvine D. J.; Alexander M. R.; Avery S. V. Discovery of (Meth)Acrylate Polymers That Resist Colonization by Fungi Associated with Pathogenesis and Biodeterioration. Sci. Adv. 2020, 6 (23), eaba6574 10.1126/sciadv.aba6574. 32548270 PMC7274803

[ref25] Crawford L. A.; Cuzzucoli Crucitti V.; Stimpson A.; Morgan C.; Blake J.; Wildman R. D.; Hook A. L.; Alexander M. R.; Irvine D. J.; Avery S. V. A Potential Alternative to Fungicides Using Actives-Free (Meth)Acrylate Polymers for Protection of Wheat Crops from Fungal Attachment and Infection. Green Chem. 2023, 25 (21), 8558–8569. 10.1039/D3GC01911J. 38013846 PMC10614722

[ref26] Zhou Z.; Ruiz Cantu L.; Chen X.; Alexander M. R.; Roberts C. J.; Hague R.; Tuck C.; Irvine D.; Wildman R. High-Throughput Characterization of Fluid Properties to Predict Droplet Ejection for Three-Dimensional Inkjet Printing Formulations. Addit. Manuf. 2019, 29, 100792 10.1016/j.addma.2019.100792.

[ref27] Brothers K. M.; Newman Z. R.; Wheeler R. T. Live Imaging of Disseminated Candidiasis in Zebrafish Reveals Role of Phagocyte Oxidase in Limiting Filamentous Growth. Eukaryot. Cell 2011, 10 (7), 932–944. 10.1128/EC.05005-11. 21551247 PMC3147414

[ref28] He Y.; Vallières C.; Alexander M. R.; Wildman R. D.; Avery S. V. Inkjet 3dprinting of Polymers Resistant to Fungal Attachment. BioProtocols 2021, 11 (9), e4016 10.21769/BioProtoc.4016. PMC8161124 34124315

[ref29] Singh T.; Hook A. L.; Luckett J.; Maitz M. F.; Sperling C.; Werner C.; Davies M. C.; Irvine D. J.; Williams P.; Alexander M. R. Discovery of Hemocompatible Bacterial Biofilm-Resistant Copolymers. Biomaterials 2020, 260, 120312 10.1016/j.biomaterials.2020.120312. 32866726 PMC7534038

[ref30] Tyler B. J.; Hook A.; Pelster A.; Williams P.; Alexander M.; Arlinghaus H. F. Development and Characterization of a Stable Adhesive Bond between a Poly(Dimethylsiloxane) Catheter Material and a Bacterial Biofilm Resistant Acrylate Polymer Coating. Biointerphases 2017, 12 (2), 02C412 10.1116/1.4984011. PMC5441992 28535686

[ref31] Wen M.; Ng L. V.; Payne J. A.; Francis L. F.; Scriven L. E.; Mccormick A. V. Kinetic Study of Free-Radical Polymerization of Multifunctional Acrylates and Methacrylates. S&T's 50th Annual Conference, 1997, pp 564–569.

[ref32] He Y.; Begines B.; Luckett J.; Dubern J.-F.; Hook A.; Prina E.; Rose F.; Tuck C.; Hague R.; Irvine D.; Williams P.; Alexander M.; Wildman R. Inkjet Based 3D Printing of Bespoke Medical Devices That Resist Bacterial Biofilm Formation, 2020. 10.1101/2020.06.30.180596.

[ref33] Choi W. C.; Gavande V.; Kim D. Y.; Lee W. K. Study on Press Formability and Properties of UV-Curable Polyurethane Acrylate Coatings with Different Reactive Diluents. Polymers 2023, 15 (4), 880 10.3390/polym15040880. 36850163 PMC9959498

[ref34] Khudyakov I. V. Fast Photopolymerization of Acrylate Coatings: Achievements and Problems. Prog. Org. Coat. 2018, 121, 151–159. 10.1016/j.porgcoat.2018.04.030.

[ref35] Okamura H.; Yamagaki M.; Nakata K. Analysis of Network Structures in Thiol-Ene UV Curing System Using Reworkable Resins. Polymers 2019, 11 (1), 5 10.3390/polym11010005. PMC6401966 30959989

[ref36] Adlington K.; Nguyen N. T.; Eaves E.; Yang J.; Chang C. Y.; Li J.; Gower A. L.; Stimpson A.; Anderson D. G.; Langer R.; Davies M. C.; Hook A. L.; Williams P.; Alexander M. R.; Irvine D. J. Application of Targeted Molecular and Material Property Optimization to Bacterial Attachment-Resistant (Meth)Acrylate Polymers. Biomacromolecules 2016, 17 (9), 2830–2838. 10.1021/acs.biomac.6b00615. 27461341 PMC6464089

[ref37] Yarusso D. J. Effect of Rheology on PSA Performance. In Adhesion Science and Engineering; Dillard D. A.; Pocius A. V.; Chaudhury M., Eds.; Elsevier Science B.V.: Amsterdam, 2002; pp 499–533.

[ref38] Reis N.; Derby B. Ink Jet Deposition of Ceramic Suspensions: Modelling and Experiments of Droplet Formation. MRS Online Proc. Libr. 2000, 625 (1), 117 10.1557/PROC-625-117.

