# Supplementary material for: Fungal Attachment-Resistant Polymers for the Additive Manufacture of Medical Devices
Source: ACS Appl Mater Interfaces. 2024 Sep 30;16(40):54509–20. doi: 10.1021/acsami.4c04833 (PMC11472319; doi:10.1021/acsami.4c04833)
Supplement: Supplementary file 1 — am4c04833_si_001.docx [file am4c04833_si_001.docx]

**Supporting Information for “Fungal Attachment Resistant Biomedical Polymers for the Additive Manufacture of Medical Devices”**

Ling Xin Yong,^a,^*^#^* Joseph Sefton,^a^ Cindy Vallières,^b,$^ Graham A. Rance,^c^ Jordan Hill,*^a^* Valentina Cuzzucoli Crucitti, ^a^ Adam A. Dundas,^d^ Felicity R.A.J. Rose,*^e^* Morgan R. Alexander, *^e^* Ricky Wildman, ^a^ Yinfeng He, ^a^ Simon V. Avery, ^b,^* Derek J. Irvine ^a,^*

*^a^ Centre for Additive Manufacturing, Department of Chemical and Environmental Engineering, University Park, University of Nottingham, Nottingham, NG7 2RD*

*^b^ School of Life Sciences, University Park, University of Nottingham, Nottingham NG7 2RD*

*^c^ Nanoscale and Microscale Research Centre, University of Nottingham, University Park, NG7 2RD*

*^d^ Advanced Materials Research Group, Department of Chemical and Environmental Engineering, University Park, University of Nottingham, Nottingham, NG7 2RD, UK*

*^e^ School of Pharmacy, University of Nottingham, Nottingham, NG7 2RD, UK*

Current affiliation

*^#^ School of Material Science & Engineering, 50 Nanyang Ave, Nanyang Technological University, Singapore 639798 and Singapore Centre for Environmental Life Sciences Engineering (SCELSE), 60 Nanyang Ave, Singapore 637551*

*^$^ CNRS, Institut de Chimie des Substances Naturelles, UPR 2301, Université Paris-Saclay, Gif-sur-Yvette, France.*

**Corresponding author emails**: [simon.avery@nottingham.ac.uk](mailto:simon.avery@nottingham.ac.uk); [derek.irvine@nottingham.ac.uk](mailto:derek.irvine@nottingham.ac.uk)


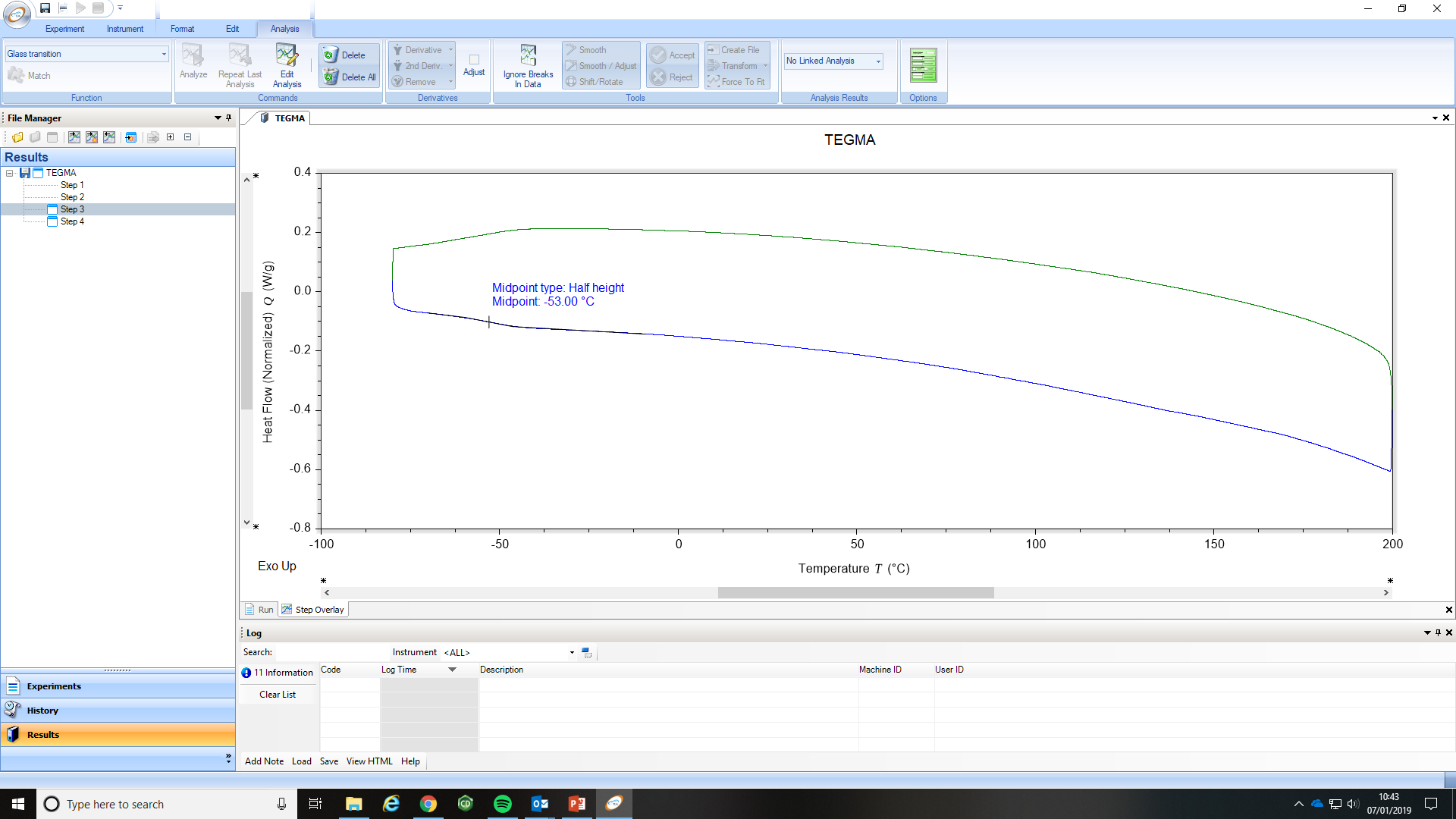


***Figure S1:*** *DSC thermogram to determine T_g_ of TEGMA homopolymer.*

*
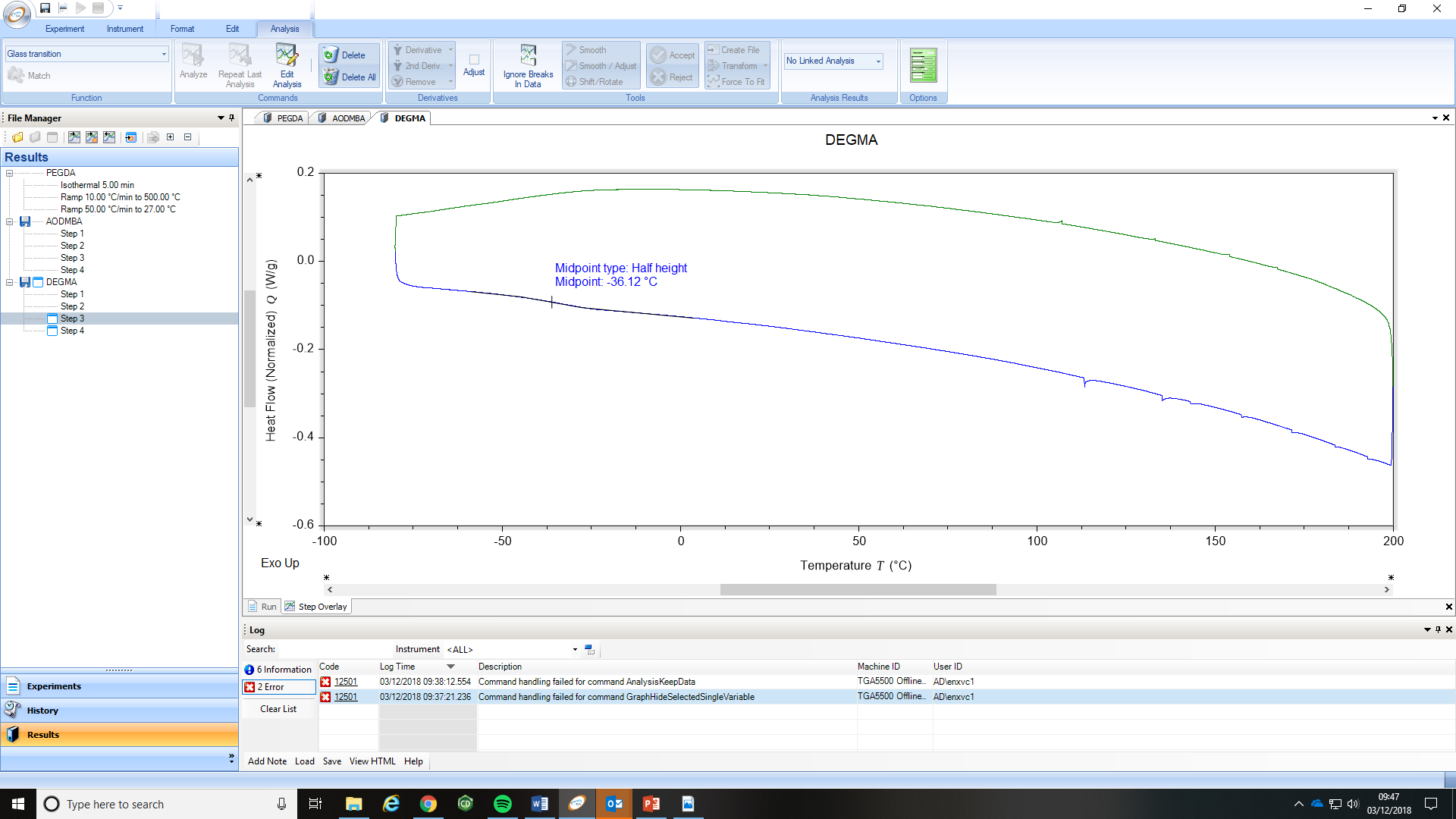
*

***Figure S2:*** *DSC thermogram to determine T_g_ of DEGMA homopolymer.*


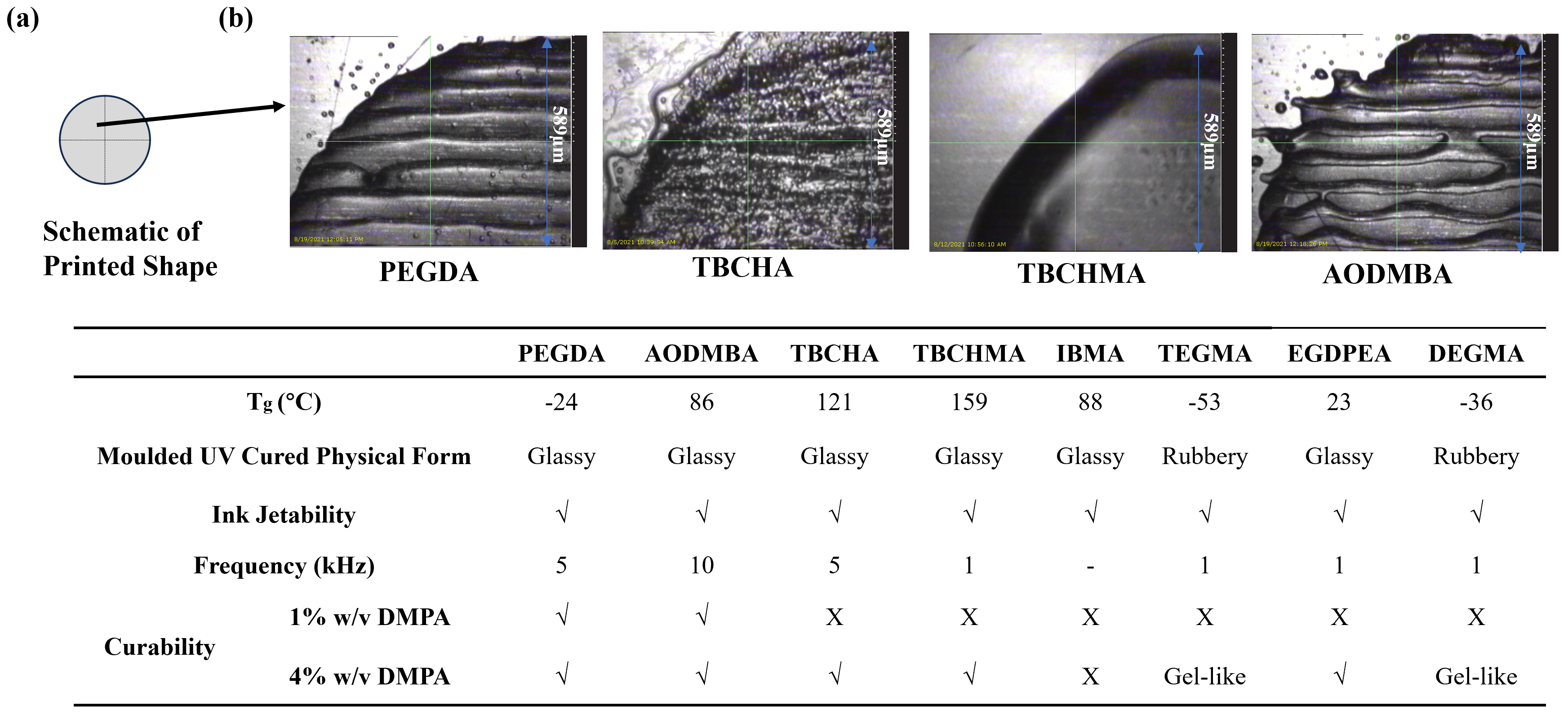


***Figure S3****: Image showing the left top quarter of 3mm circular discs printed as a two-layer print using four of the chosen monomers with a 4% w/v DMPA photo-initiator loading taken by fiducial camera of the Dimatix DMP-2830 where PEGDA, TBCHA, TBCHMA, and AODMBA were able to form solid stable layers,*

***
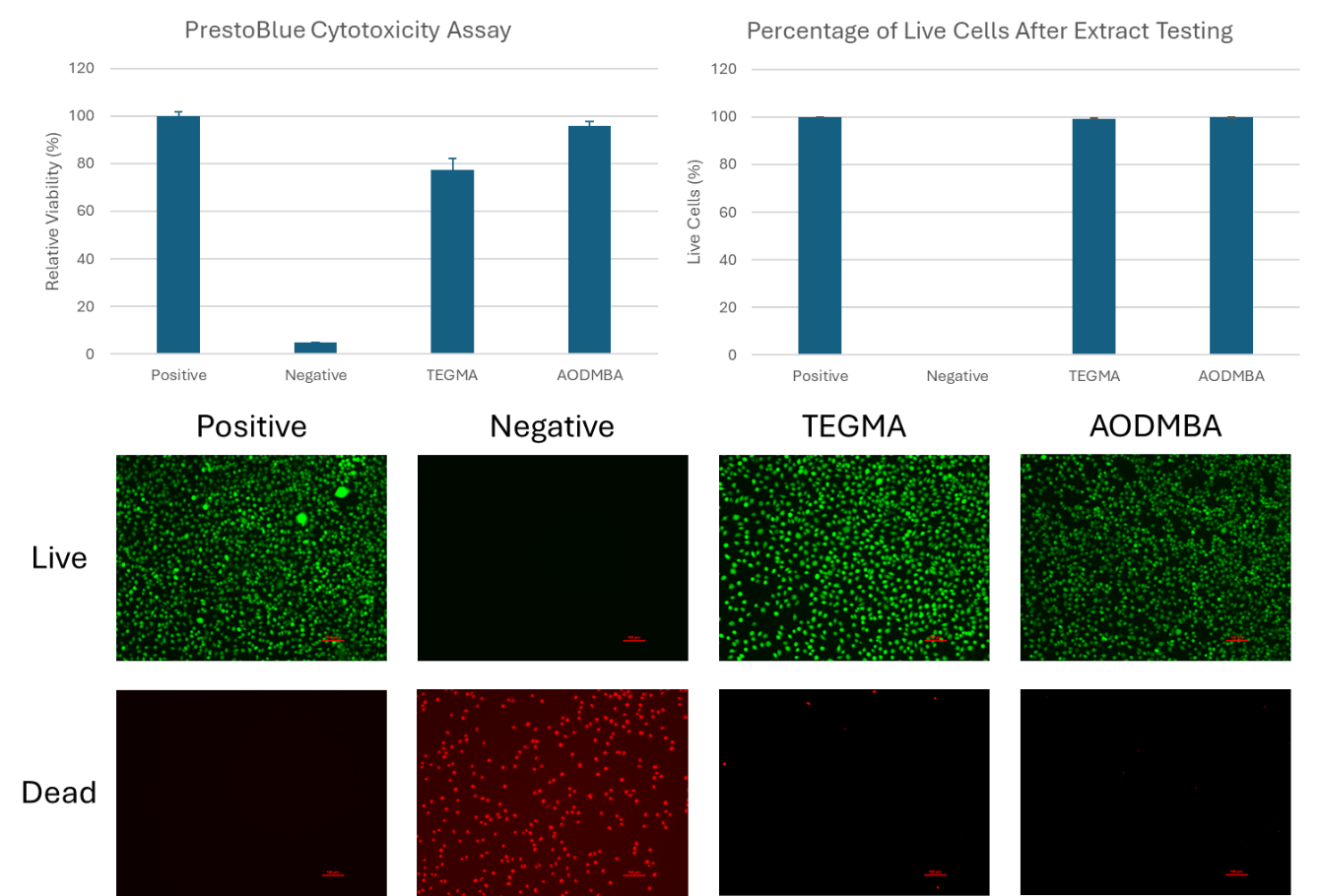
***

***Figure S4:*** *Extract cytotoxicity testing demonstrating the viability of L929 mouse fibroblasts when cultured for 24 hours in the presence of extract media from the homopolymers TEGMA and AODMBA compared to a negative (DMSO) and positive control (tissue culture plastic). Scale = 100 µm.*


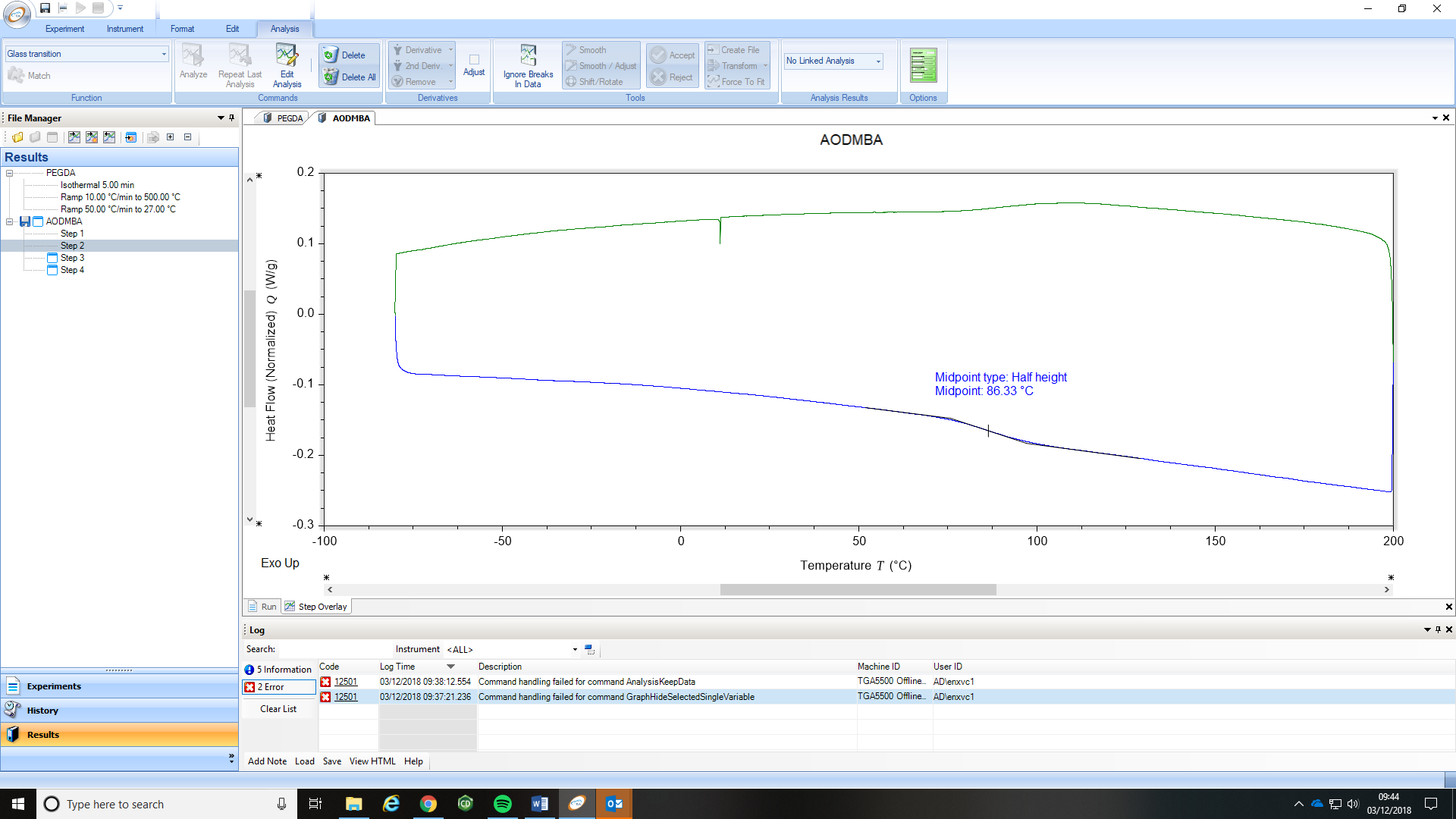


***Figure S5:*** *DSC thermogram to determine T_g_ of AODMBA homopolymer.*

*
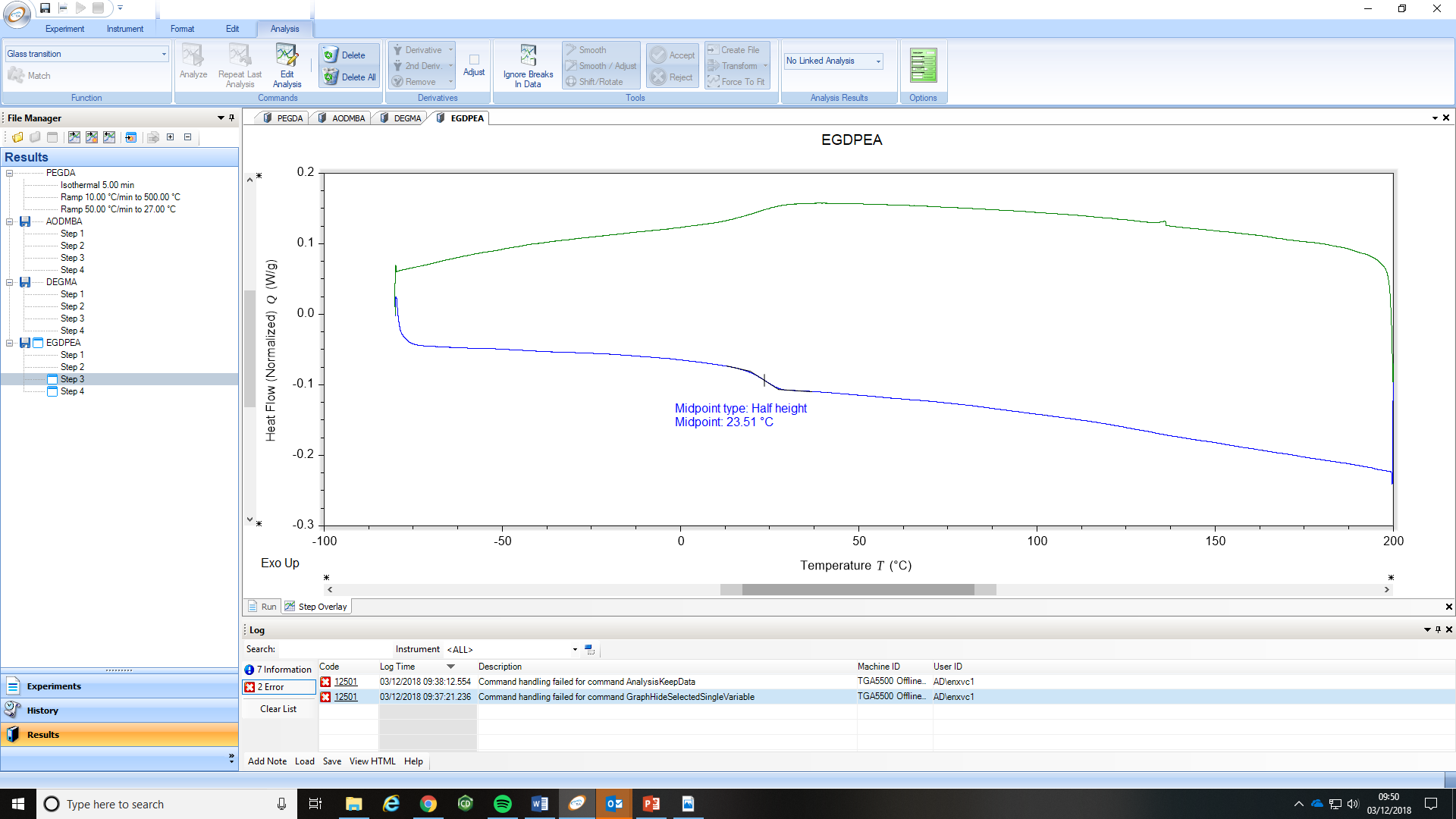
*

***Figure S6:*** *DSC thermogram to determine T_g_ of EGDPEA homopolymer.*


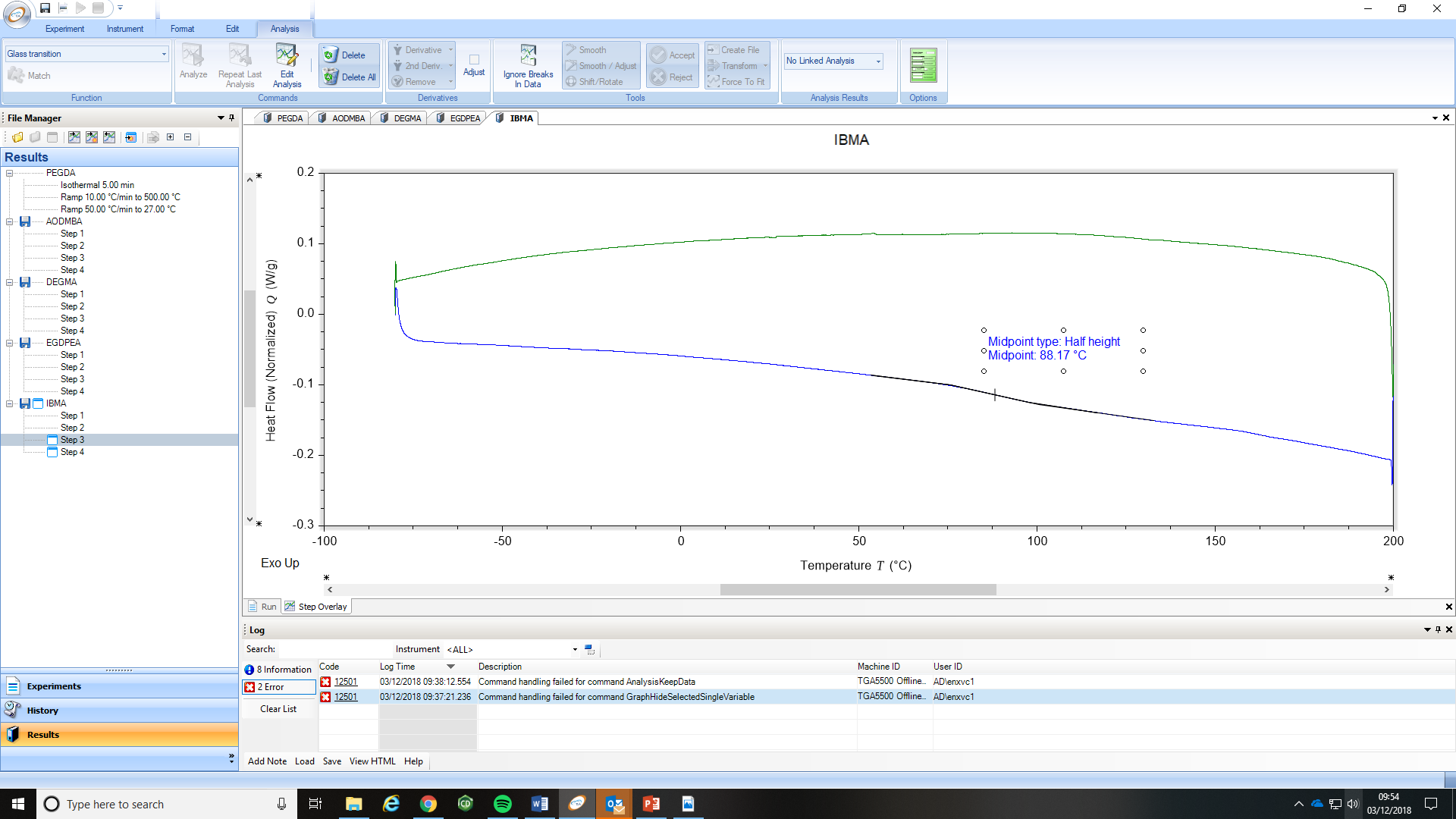


***Figure S7:*** *DSC thermogram to determine T_g_ of IBMA homopolymer.*

*
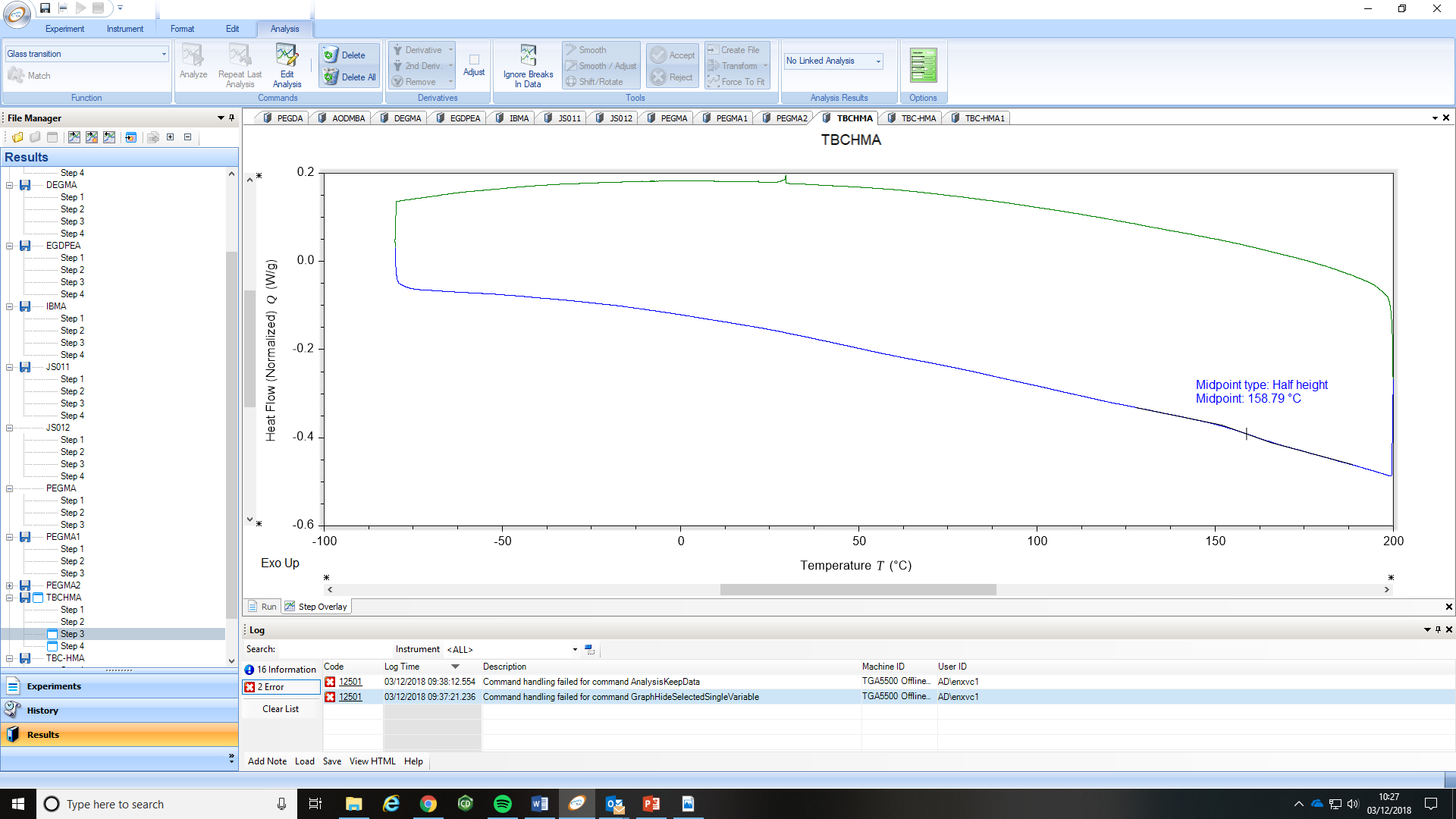
*

***Figure S8:*** *DSC thermogram to determine T_g_ of TBCHMA homopolymer.*


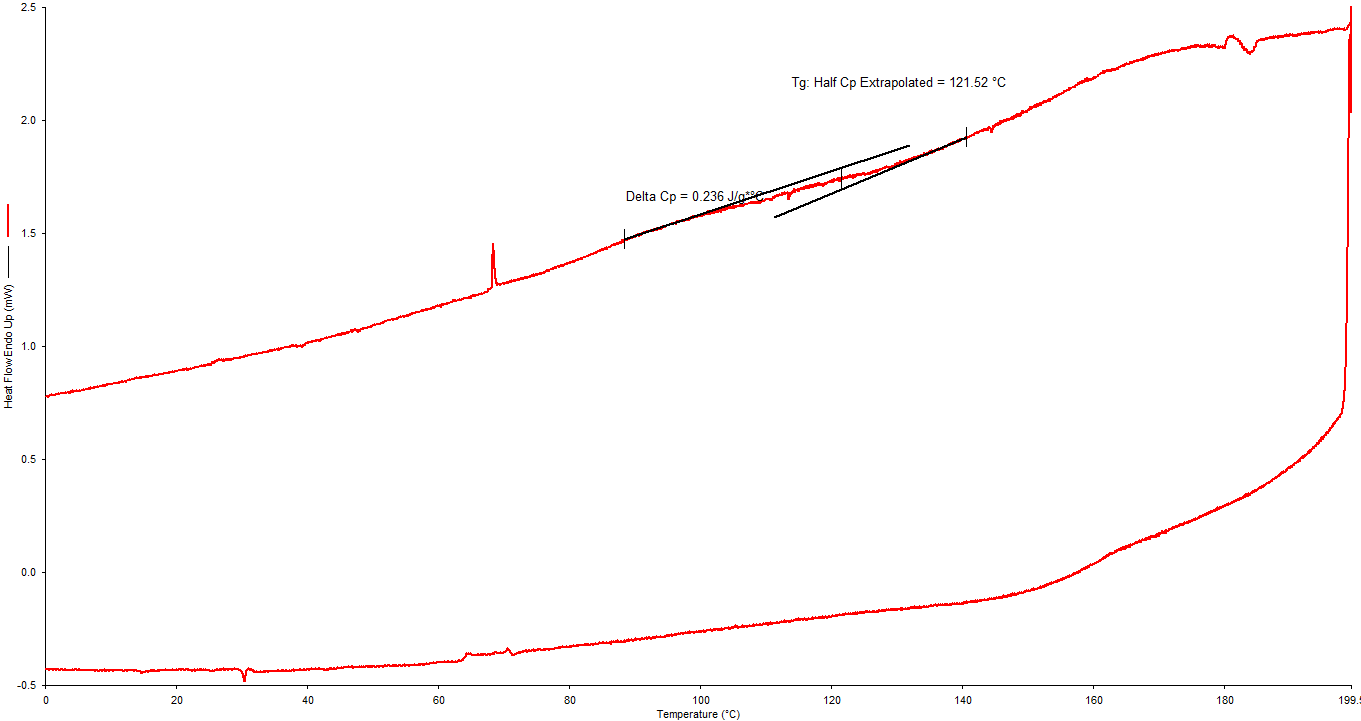


***Figure S9:*** *DSC thermogram to determine T_g_ of TBCHA homopolymer.*


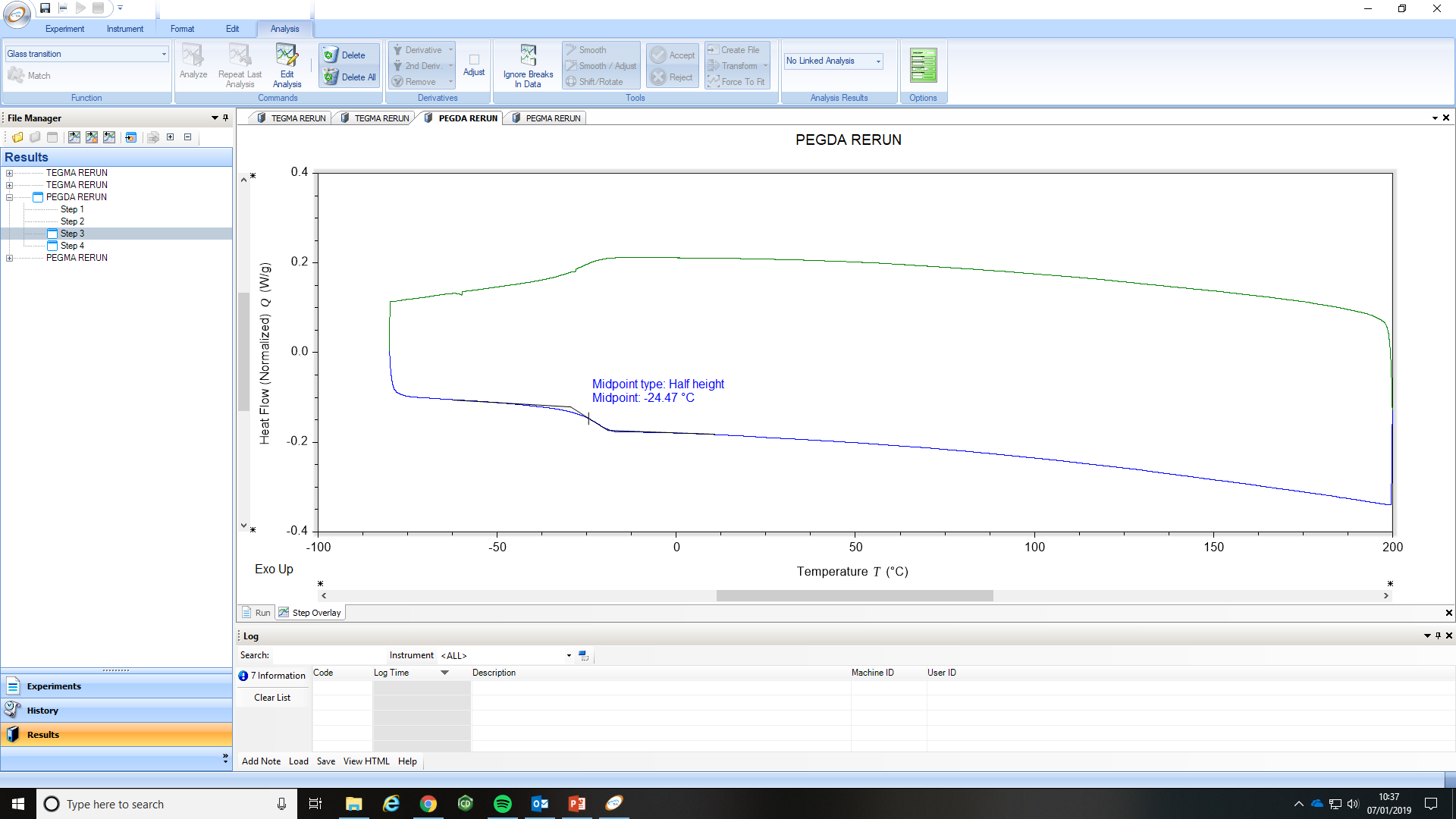


***Figure S10:*** *DSC thermogram to determine T_g_ of PEGDA homopolymer.*


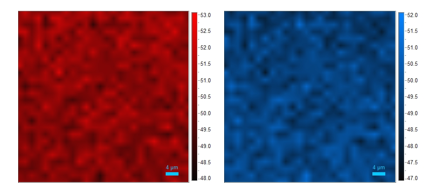


***Figure S11:*** *False color images obtained by Raman spectroscopy analysis of TEGMA:AODMBA_75:25_ showing the spatial distribution of TEGMA (red) and AODMBA (blue). The intensity axes describe the score obtained by CLS analysis of the multispectral data set.*


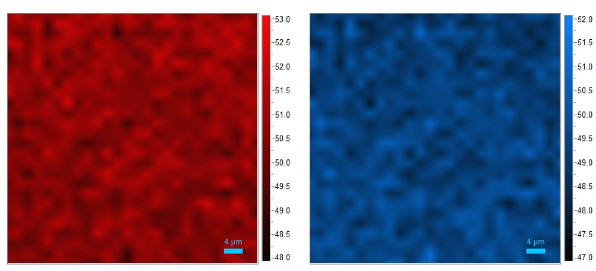


***Figure S12:*** *False color images obtained by Raman spectroscopy analysis of TEGMA:AODMBA_50:50_ showing the spatial distribution of TEGMA (red) and AODMBA (blue). The intensity axes describe the score obtained by CLS analysis of the multispectral data set.*


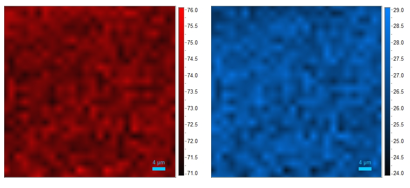


***Figure S13:*** *False color images obtained by Raman spectroscopy analysis of TEGMA:AODMBA_25:75_ showing the spatial distribution of TEGMA (red) and AODMBA (blue). The intensity axes describe the score obtained by CLS analysis of the multispectral data set.*


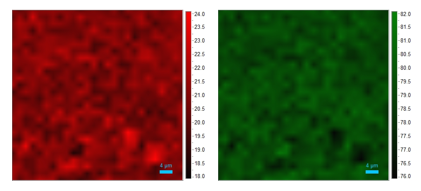


***Figure S14:*** *False color images obtained by Raman spectroscopy analysis of TEGMA:TBCHA_75:25_ showing the spatial distribution of TEGMA (red) and TBCHA (green). The intensity axes describe the score obtained by CLS analysis of the multispectral data set.*


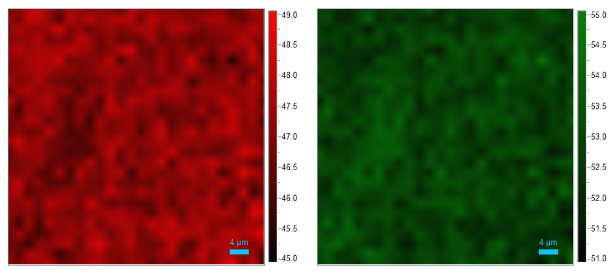


***Figure 15:*** *False color images obtained by Raman spectroscopy analysis of TEGMA:TBCHA_50:50_ showing the spatial distribution of TEGMA (red) and TBCHA (green). The intensity axes describe the score obtained by CLS analysis of the multispectral data set.*


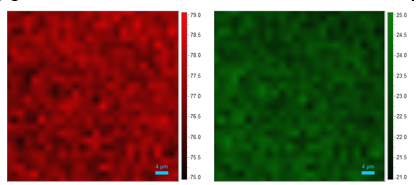


***Figure 16:*** *False color images obtained by Raman spectroscopy analysis of TEGMA:TBCHA_25:75_ showing the spatial distribution of TEGMA (red) and TBCHA (green). The intensity axes describe the score obtained by CLS analysis of the multispectral data set.*

***Table S1:*** *Room temperature viscosity and surface tension data (three replicates for each) for the two comonomers (prepared using 4% w/v DMPA initiator). These data were required to evaluate its actual ink jetability by calculating its Z parameter.*

| ***Comonomer inks*** | ***Viscosity @ RT (mPa.s)*** | ***Std. Dev. Viscosity***  ***(± mPa.s)*** | ***Surface Tension (mN/m)*** | ***Std. Dev. Surface Tension***  ***(± mN/m)*** |
| --- | --- | --- | --- | --- |
| *TEGMA:AODMBA_50:50_* | *9.13* | *0.27* | *34.03* | *0.86* |
|  | *9.42* |  | *32.84* |  |
|  | *8.89* |  | *33.50* |  |
| *TEGMA:TBCHA_50:50_* | *4.94* | *0.20* | *33.19* | *1.71* |
|  | *4.58* |  | *34.80* |  |
|  | *4.91* |  | *31.39* |  |


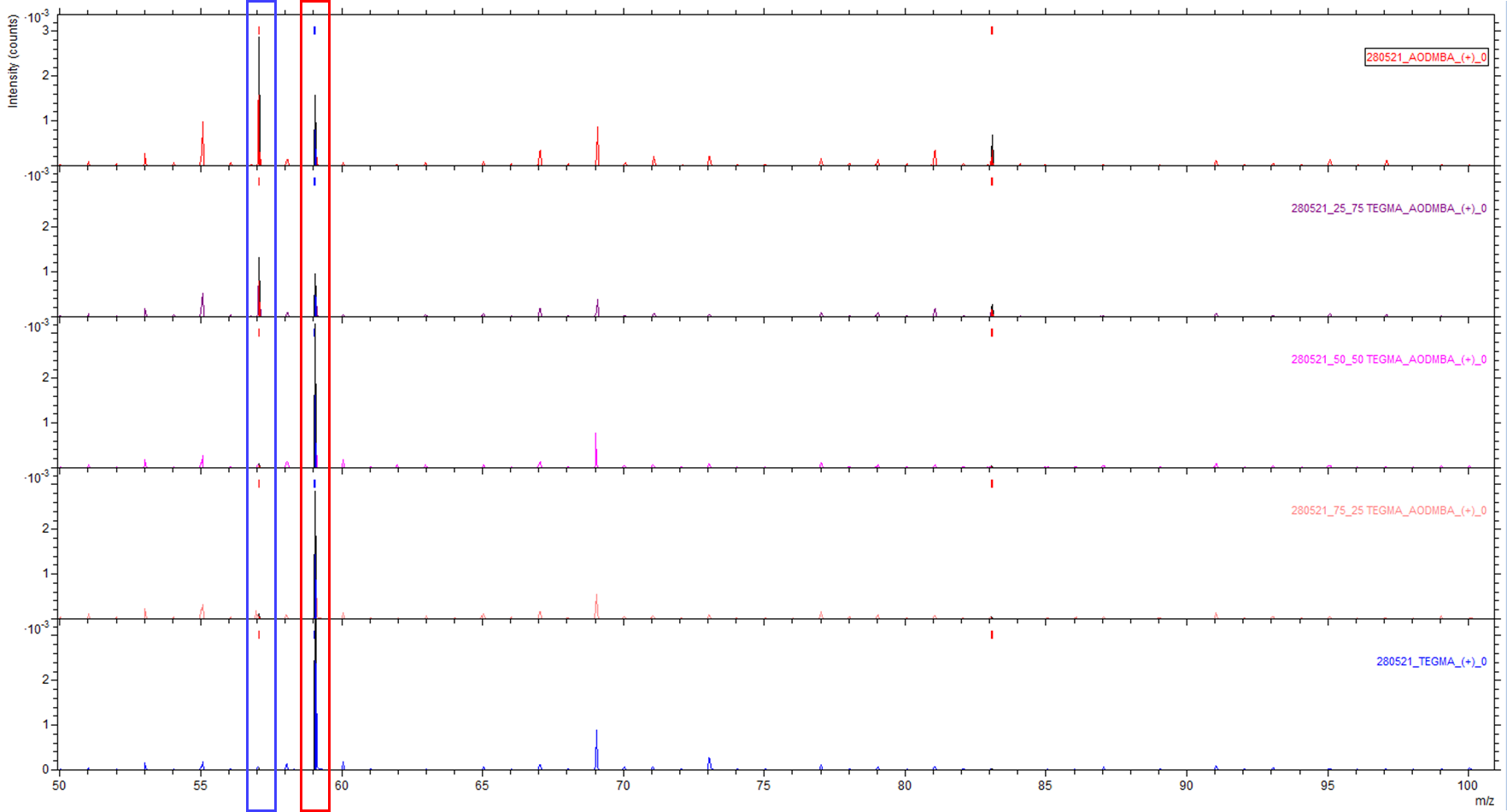


***Figure S17:*** *Positive ToF-SIMS overlap data of AODMBA:TEGMA series (m/z 50-100) showing assignment of C_4_H_9_^+^ (blue) to AODMBA and C_4_H_7_O^+^ (red) to TEGMA.*

*
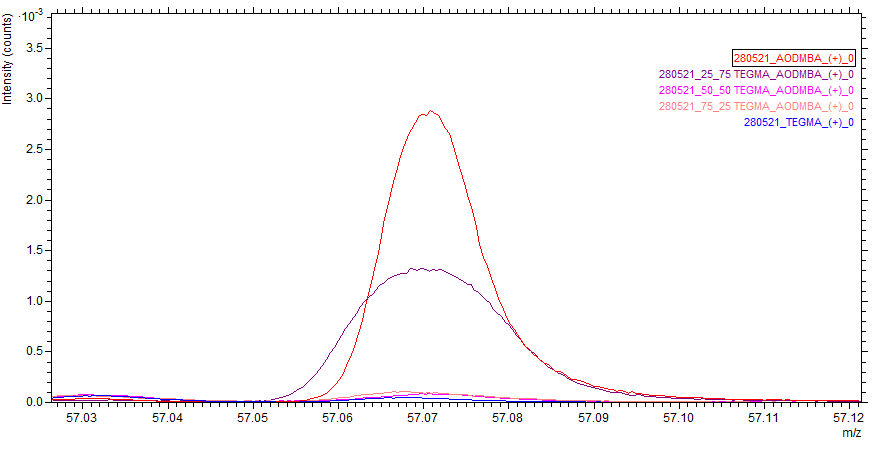
*

***Figure S18:*** *Positive ToF-SIMS overlap data of AODMBA:TEGMA at 57u showing peaks for materials associated to AODMBDA:TEGMA copolymer series*


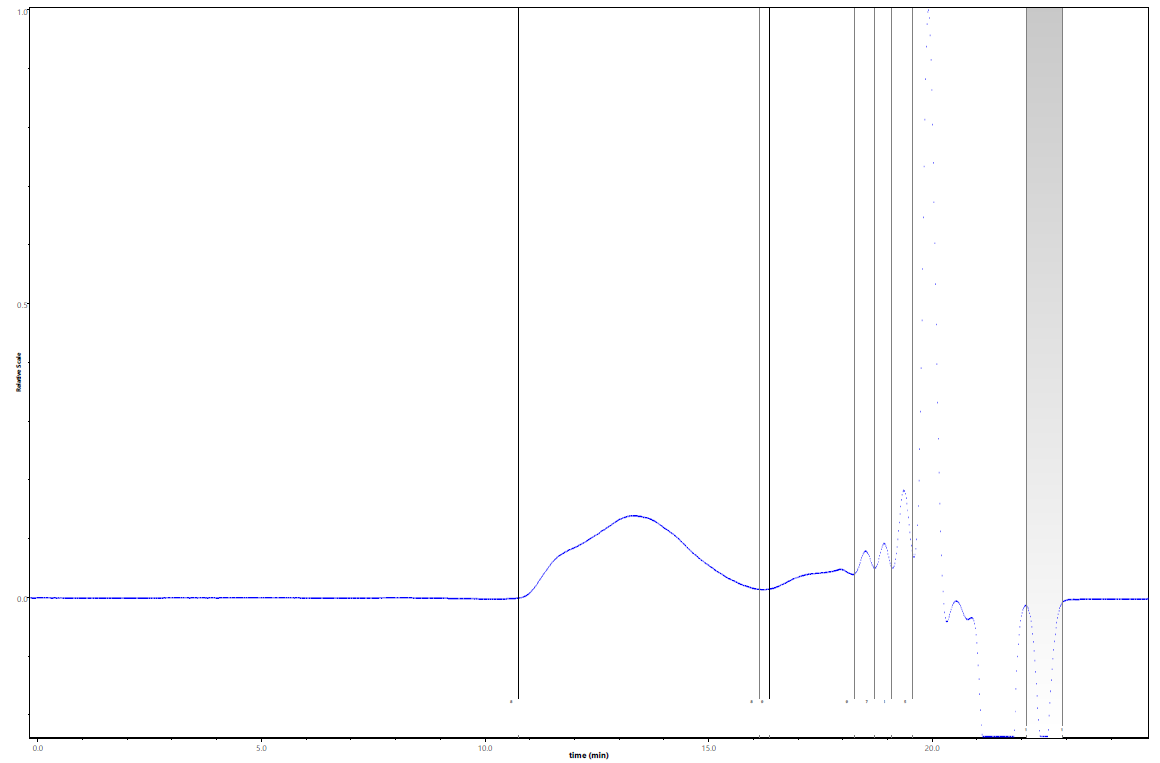


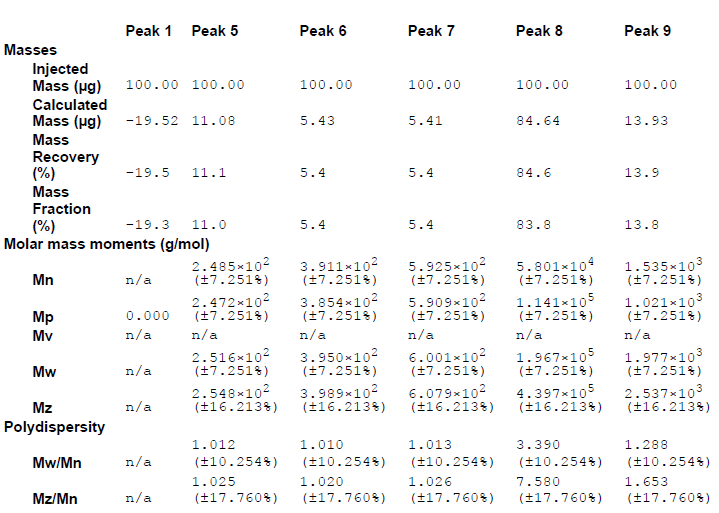


***Figure S19:*** *GPC chromatogram of TEGMA:AODMBA_35:65_.*


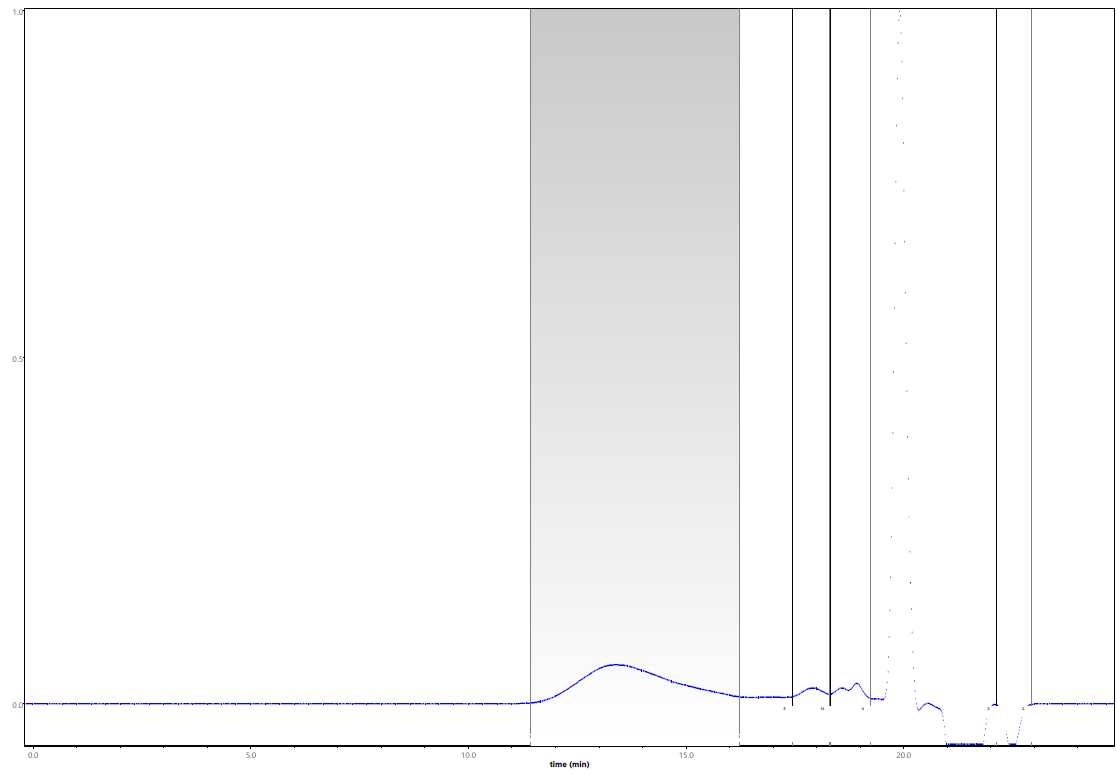


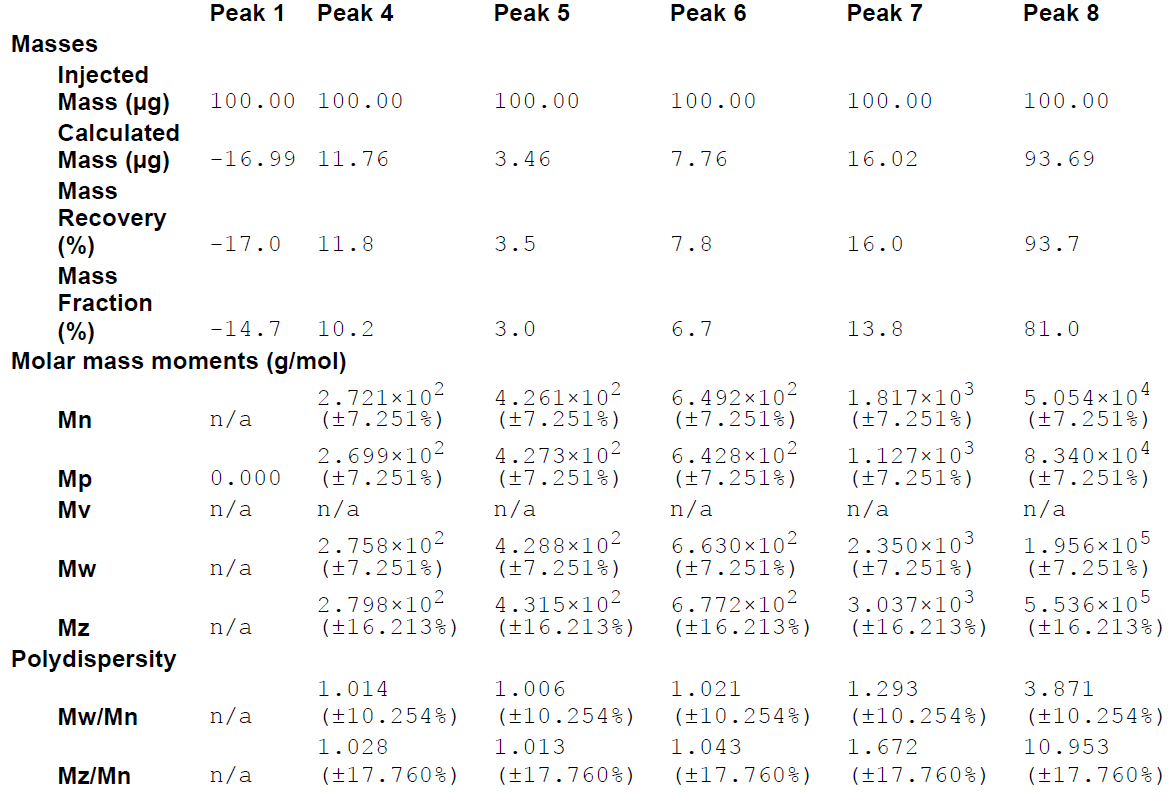


***Figure S20:*** *GPC chromatogram of TEGMA:AODMBA_40:60_.*


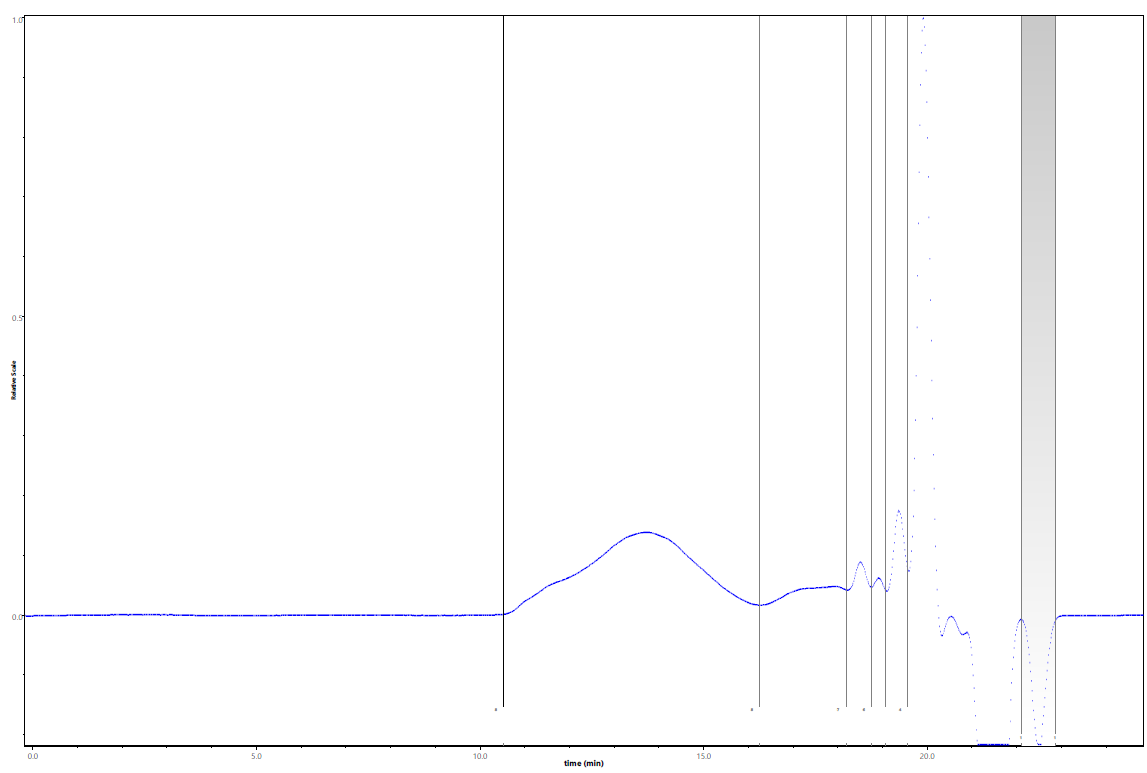

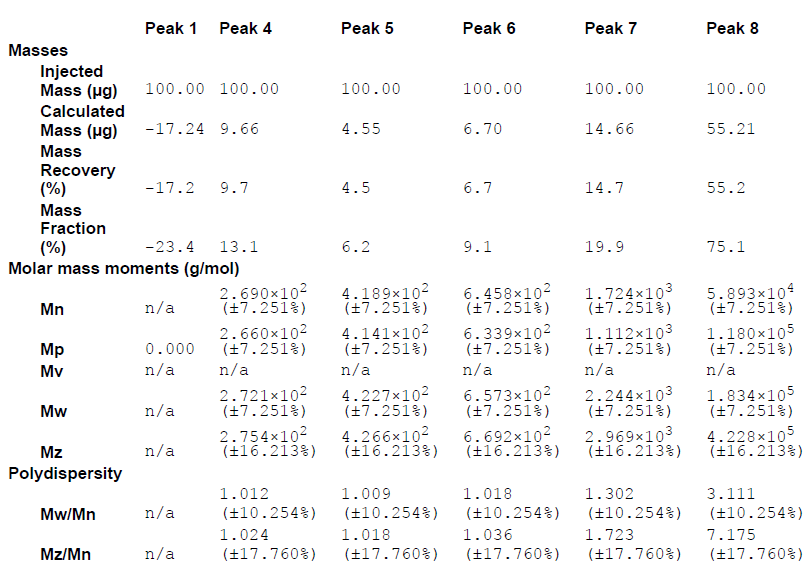


***Figure S21:*** *GPC chromatogram of TEGMA:AODMBA_50:50._.*


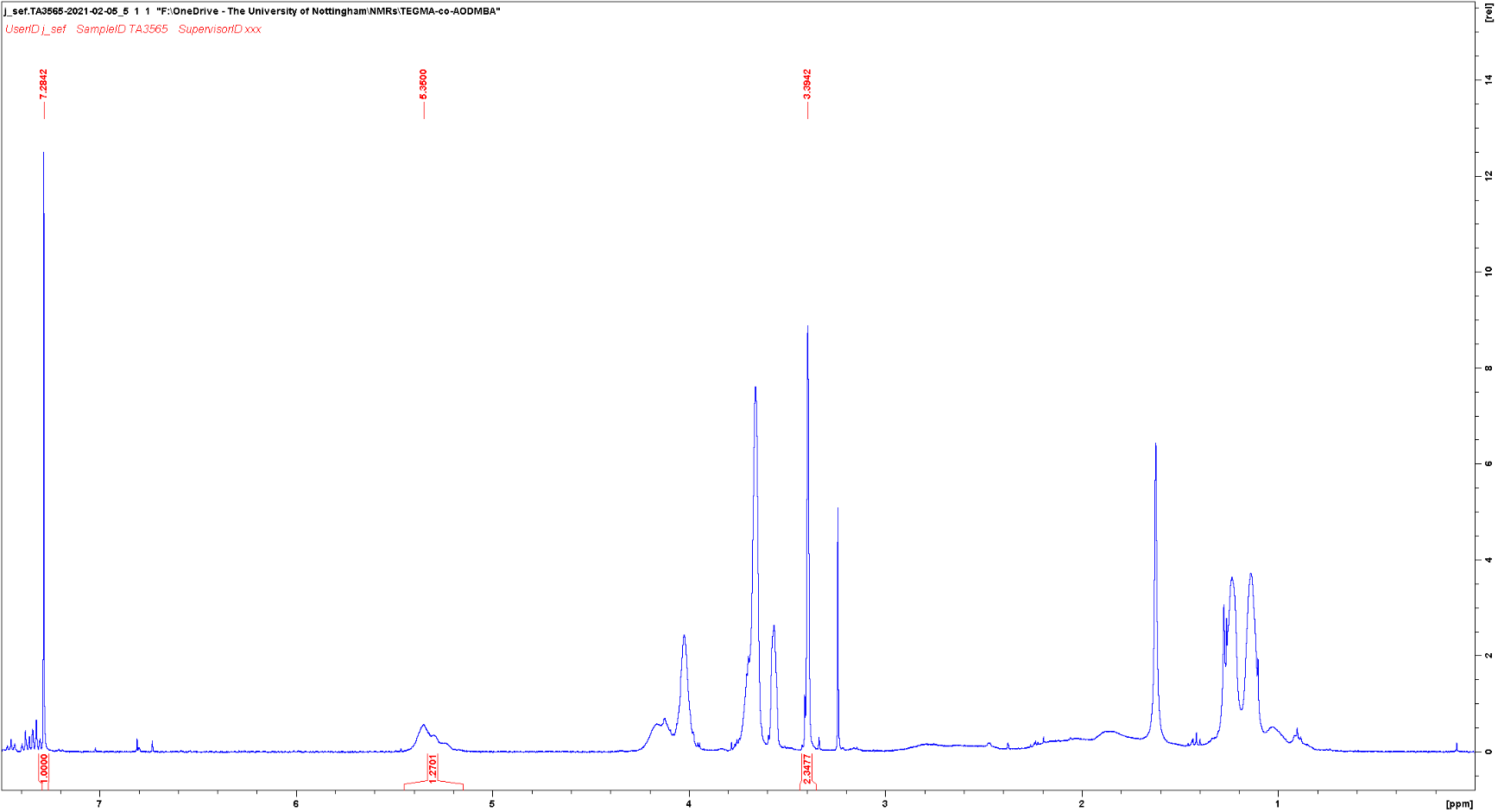


***Figure S22:*** *^1^H NMR spectrum of TEGMA:AODMBA_35:65._.*


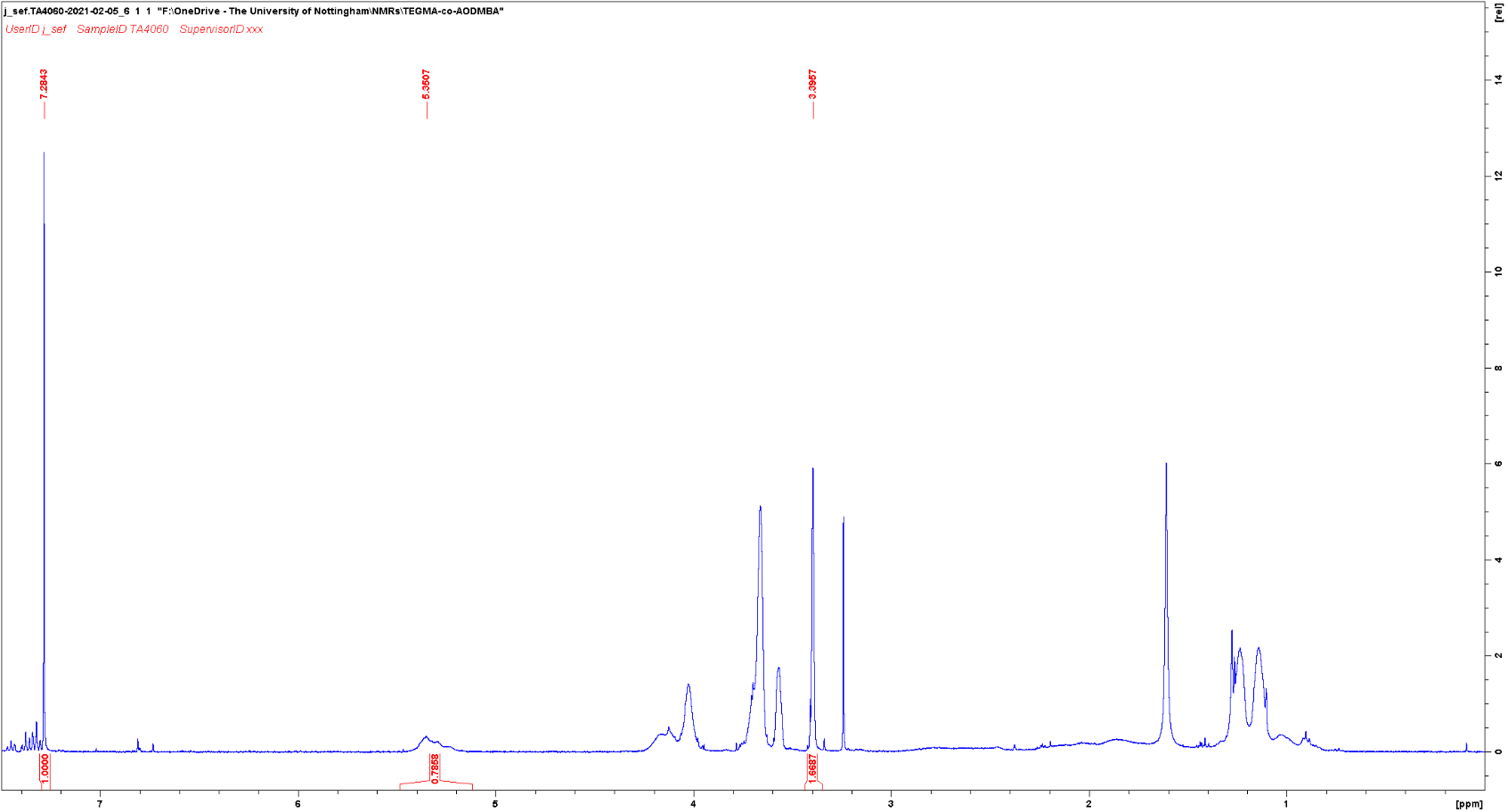


***Figure S23:*** *^1^H NMR spectrum of TEGMA:AODMBA_40:60._*


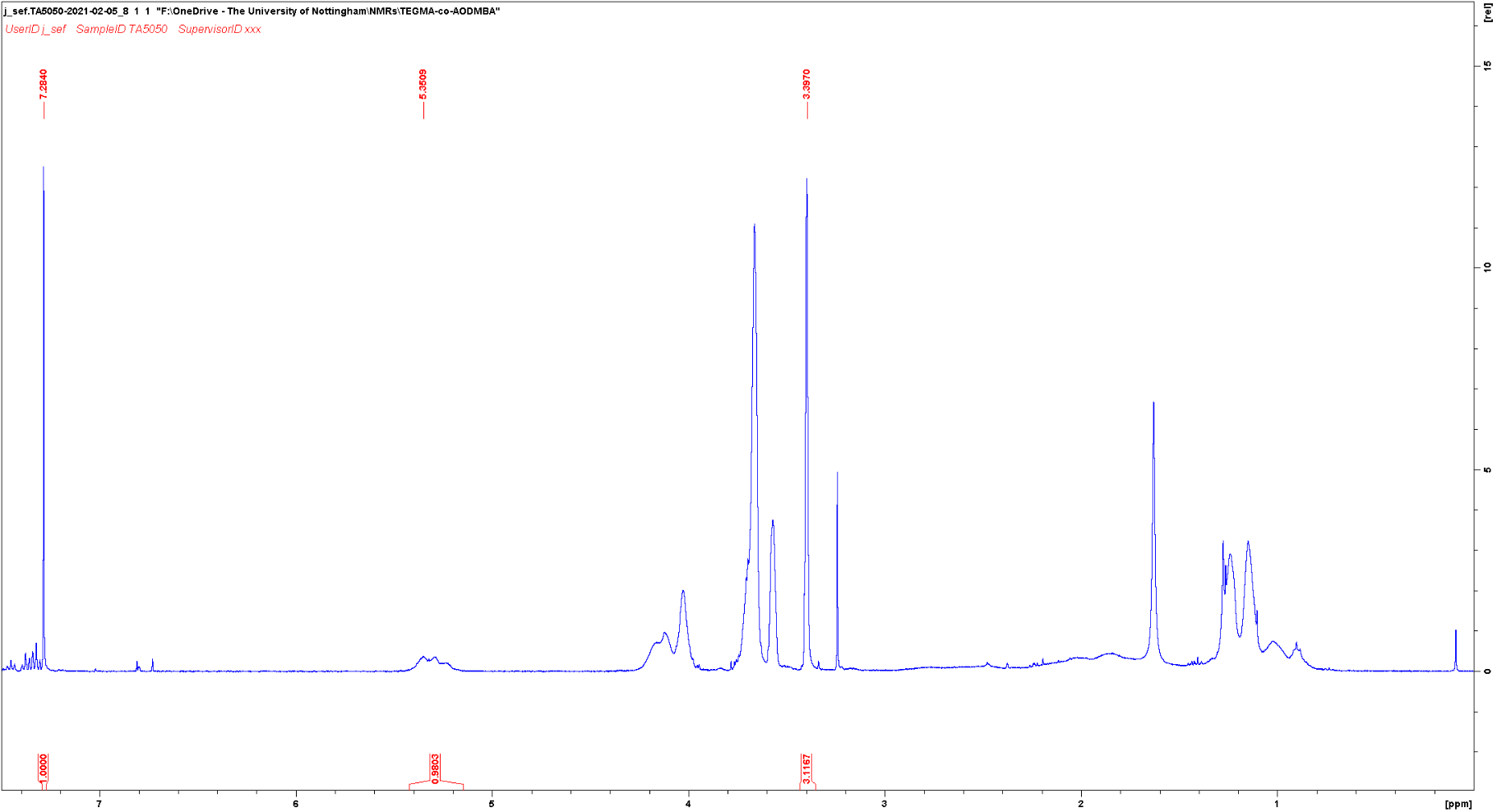


***Figure S24:*** *^1^H NMR spectrum of TEGMA:AODMBA_50:50._.*


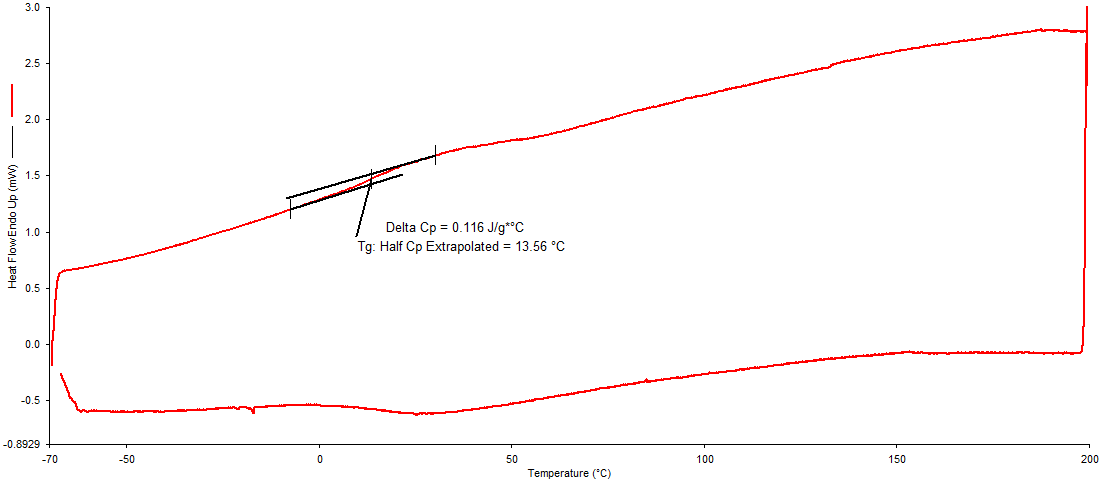


***Figure S25:*** *DSC thermogram to determine T_g_ of TEGMA:AODMBA_40:60_ copolymer.*
